# DENV-2 3′UTR dumbbell structure is a critical factor for viral infection and dissemination in *Aedes* mosquito

**DOI:** 10.1128/jvi.00758-25

**Published:** 2025-07-22

**Authors:** Xiaoyu Wang, Ying Huang, Fei Wang, Qianyun Hu, Doudou Huang, Haixia Ma, Yi Liu, Qiyong Liu, Bo Zhang, Zhiming Yuan, Han Xia

**Affiliations:** 1 Key Laboratory of Virology and Biosafety, Wuhan Institute of Virology, Chinese Academy of Sciences 74614 , Wuhan, Hubei, China; 2 University of the Chinese Academy of Sciences 74519, Beijing, China; 3 Hubei Jiangxia Laboratory, Wuhan, Hubei, China; 4 National Key Laboratory of Intelligent Tracking and Forecasting for Infectious Diseases, National Institute for Communicable Disease Control and Prevention, Chinese Center For Disease Control and Prevention 96698, Beijing, China; Wake Forest University School of Medicine, Winston-Salem, North Carolina, USA

**Keywords:** flaviviruses, DENV, 3'UTR dumbbell structure, *Aedes* mosquito, dissemination

## Abstract

Dengue virus (DENV) is a mosquito-borne flavivirus with the capability of replicating and spreading between vertebrate and invertebrate hosts, which poses a huge threat to global public health. Understanding the molecular mechanism of its replication and transmission in mosquitoes is crucial for controlling the dengue epidemic. The 3′ untranslated region (UTR) has been identified as a mediator for viral replication, pathogenicity, and transmission, but its effect on DENV in the mosquito host remains unknown. This study investigated the role of the dumbbell (DB) structure within the 3′UTR of DENV-2 during mosquito infection. The results showed that the absence of the DB structure reduced DENV-2 viral replication efficiency in both mosquito cell lines and *Aedes* mosquito adults. The DB structure is essential for DENV-2 to escape from the midgut infection and scape barriers, but not for the salivary gland infection and escape barriers. The transcriptome analysis also revealed that the absence of the DB structure increased the expression of Defensin A and Defensin C, which can inhibit viral replication in midgut and spread to hemolymph at 7–10 days post-infection. Through siRNA-mediated knockdown and overexpression experiments in C6/36 cells, we further established that Defensin C exhibits particularly inhibitory effects on the absence of DB structure DENV-2. Our findings highlight the critical role of the DB structure in DENV-2 adaptation to the mosquito host and provide insights into the molecular mechanisms underlying flavivirus transmission, contributing to the understanding of flavivirus-host interactions and assisting the development of strategies for controlling dengue fever.

**IMPORTANCE:**

Dengue virus (DENV) transmitted by Aedes mosquitoes threatens global health. This study focuses on the dumbbell (DB) structure in DENV-2’s 3′ untranslated region, which is crucial for viral replication and transmission in mosquitoes. We discovered that the absence of the DB structure reduces DENV-2’s replication efficiency and impairs its ability to evade the mosquito midgut barrier. Transcriptome data suggest that the DB structure may facilitate viral escape by modulating the expression of Defensin A and Defensin C. Functional validation in C6/36 cells demonstrates that Defensin C acts as an inhibitor, suppressing the replication of DENV-2 with the absence of DB structure and revealing a novel RNA structure-mediated immune evasion strategy. The findings of this study highlight the critical role of the DB structure in DENV-2 adaptation to mosquito hosts and provide valuable insights into the mechanisms of flavivirus transmission and potential strategies for dengue control.

## INTRODUCTION

Mosquito-borne flaviviruses (MBFVs), such as dengue virus (DENV), Japanese encephalitis virus (JEV), Zika virus (ZIKV), and yellow fever virus (YFV), all belonging to the genus *Orthoflavivirus*, can cause disease and mortality in both humans and animal hosts (1–4). Dengue has seen a remarkable surge in incidence, with an estimated annual occurrence of 100–400 million DENV infections (WHO, 2024), imposing a substantial burden on global public health.

Transmission of MBFVs occurs through different mosquito vectors. Where DENV and ZIKV are mainly transmitted through *Aedes* mosquitoes, JEV and West Nile virus (WNV) are transmitted mainly through *Culex* mosquitoes (5). During the virus cycle among mosquito vectors and vertebrate hosts, MBFVs use specific strategies to adapt to the antiviral response of different hosts, facilitating virus replication and transmission (6). It has been reported that 3′UTRs of MBFVs play a crucial role in viral adaptation to different hosts (7, 8).

The 3′UTR in MBFVs ranges from 400 to 700 nucleotides (nt) in length and is primarily divided into three distinct domains I, II, and III based on conserved secondary structures. Domain I is situated downstream of the stop codon and encompasses stem-loop (SL) structures (9). Domain II contains dumbbell (DB) structures and is conserved across *Orthoflavivirus* (9, 10). The number of SL and DB structures varies among different MBFVs (11). Domain III is located at the distal end of the 3′UTR, exhibiting a small hairpin (sHP) and a terminal 3′ SL among MBFVs (10). The top loop sequence of the SL and DB structure complements the downstream sequence to form a pseudoknot (PK), which is a more stable tertiary structure (12). This PK is vital for preventing degradation by host 5′−3′ exoribonuclease Xrn1 and facilitating the production of subgenomic flavivirus RNAs (sfRNAs) (6, 13).

In a vertebrate host, the point mutation in SLII caused highly attenuated ZIKV replication in IFN-competent human cell lines A549, BeWo, and HTR-8 (14). Disrupting the DB1 structure of ZIKV decreased caspase-3 activation and mitigated cellular pathology in A549 cells (15). The absence of SLII and SLIV impacted WNV replication efficiency in Vero cells and weakened WNV pathogenicity in mice (16, 17). The changes in JEV SLIV and DB structures enhanced viral replication in BHK-21 cells, while simultaneously reducing pathogenicity and neuronal invasion in BALB/c mice (18, 19).

Understanding the elements that impact mosquito infection and transmission is also crucial. Point mutations in SLII, SLIV, and DB1 structure reduced the replication of WNV in C6/36 cells (17). The modification of SLII’s top ring sequence affected the transmission of WNV in *Culex pipiens pallens* mosquitoes by impacting infection rates in the midgut and saliva (20). Point mutations in ZIKV SLI and SLII decreased the transmission efficiency in *Ae. aegypti* mosquitoes, and this was also observed when the top ring sequence of SLI was altered (21, 22).

For DENV, the SL plays a crucial role in preserving DENV-2’s heightened adaptability during its switching between mosquito and mammalian hosts. Mutations in SLI don’t impact on DENV-2 replication in mosquito cells C6/36, while in mammalian (A549 and BHK), viral replication showed an approximately threefold reduction (11). In contrast, mutations and deletions of SL-II enhanced viral replication in C6/36 cells but attenuated in mammalian cells (11). The adaptive mutations in the 3′UTR front end and SLI of DENV-2 can also hinder the Toll immune pathway in *Ae. aegypti’*s salivary glands to enhance the infection rate in saliva (23).

The DB structures also play a crucial role in the dual-host adaptation of DENV-2 simultaneously. Following five passages of human-adapted DENV in mosquito cell lines, significant enrichment of sfRNA1, sfRNA3, and sfRNA4 was observed (7). The accumulation of sfRNA3 and sfRNA4 is significantly correlated with the DB structures, but the precise underlying mechanism remains unclear (7). It was recently shown that the top loop sequence of DENV-2 DB2 structure and the core cyclization element engage in competition, while the absence of DB2 structure facilitates virus replication in mosquito cell lines (4, 13). Although these studies improved the understanding of the DB structure for DENV replication and dual-host adaptation, its role in viral infection and spread in mosquitoes remain unexplored.

Our previous study found that the deletion of both DB structures of DENV-2 (D2Y98P/∆DB1-2) could reduce the viral replication and cytopathogenicity in vertebrate cell lines, possibly by regulating the phosphatidylinositol 3-kinase (PI3K)/Akt signaling pathway through a Bcl-2-related mechanism, ultimately causing apoptotic cell death (24, 25). In this study, we employed the DB-absent DENV-2 to investigate the role of the DB structure in the mosquito host. Through comparative infection experiment spanning mosquito cells (C6/36 and Aag2), vertebrate cells (BHK-21 and Vero E6), and *Aedes* mosquito adults, coupled with transcriptomic profiling and targeted functional validation experiments, we revealed that the DB structure may facilitate DENV-2 escape from the midgut by modulating immune gene Defensin C (DefC). The findings will provide new insights into how the DB structure contributes to the viral infection and transmission of DENV in the mosquito.

## RESULTS

### Construction for DB-deficient DENV-2

**Fig 1:**
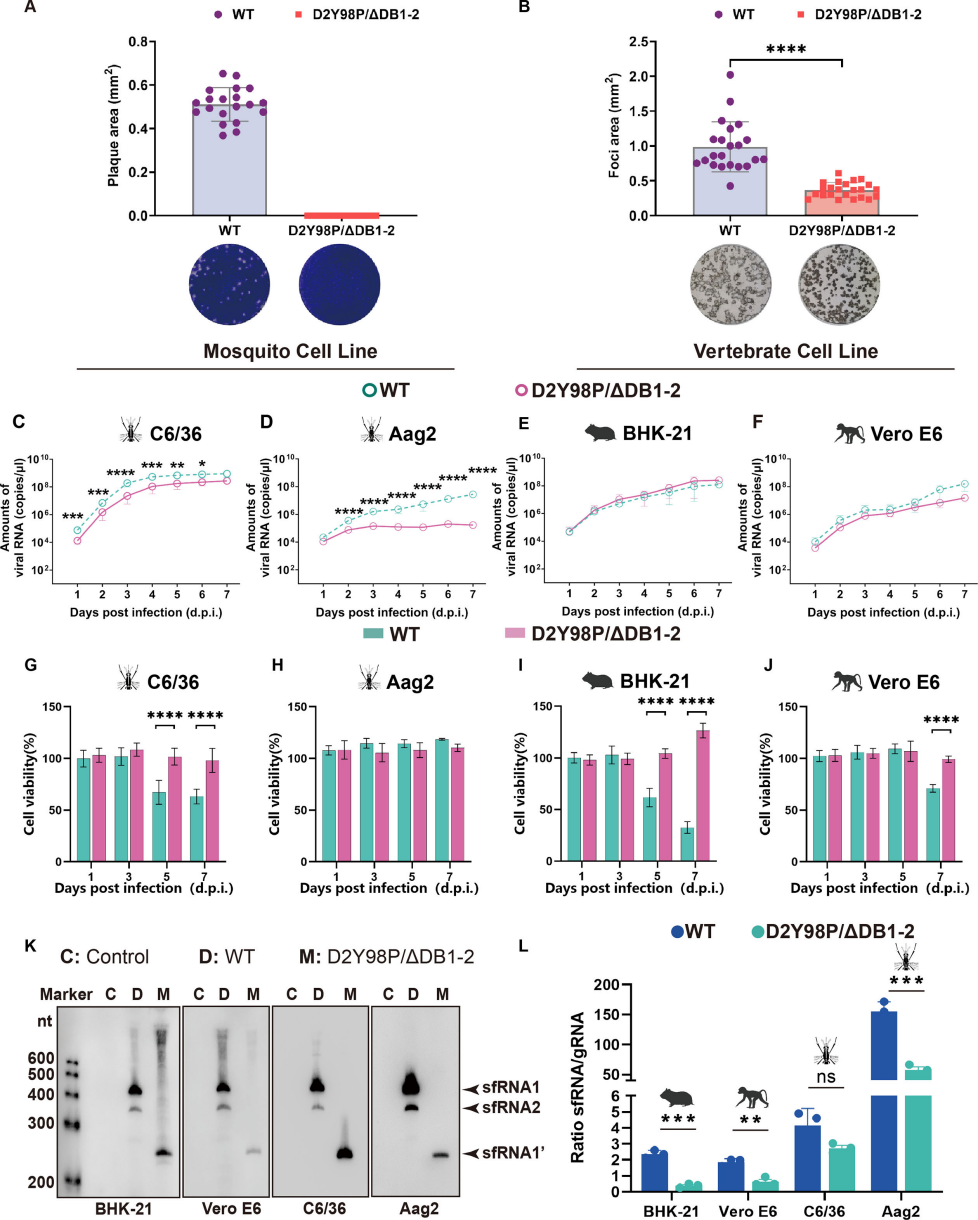
Impact of the DB structure for DENV-2 on cytotoxicity, viral replication, and sfRNA production in mosquito and vertebrate cell lines. (**A**) Plaque morphology and (**B**) immunostaining focus formation of WT and D2Y98P/ΔDB1-2 in BHK-21 cells at 4 days post-infection (d.p.i.). Quantification of mean plaque/foci areas (mm²) was performed using ImageJ (v1.54) with individual measurements represented as scatter points (data from three independent biological replicates, *n* = 9 technical replicates). (**C–F**) Viral replication kinetics in mosquito cell lines (**C**) C6/36 and (**D**) Aag2 and vertebrate cell lines (**E**) BHK-21 and (**F**) Vero E6 infected at multiplicity of infection (MOI) of 0.01. The cell supernatant was collected at every 24 h until 7 d.p.i., as detected by qRT-PCR. Green and red trajectories represent WT and D2Y98P/ΔDB1-2, respectively, with symbols indicating mean ± SD from two biological replicates (*n* = 6 technical replicates). (**G–J**) The effects of DB structure on cytotoxicity measured by CCK-8 assay in mosquito cell lines (**G**) C6/36 and (**H**) Aag2 and vertebrate cell lines (**I**) BHK-21 and (**J**) Vero E6 infected at MOI = 0.01 with WT shown in green column and D2Y98P/ΔDB1-2 in red column. Columns depict mean viability ± SD from three biological replicates (*n* = 18 technical replicates). (**K**) Northern blot analysis of sfRNA production in different cell lines (MOI = 1). Total RNA isolated from infected cells at 72 h post-infection. The experiment was repeated three times. (**L**) Ratios of sfRNA/gRNA detected by qRT-PCR in WT (blue) or D2Y98P/ΔDB1-2 (green) with infection of different cell lines. All data expressed as the mean ± SD, and bars represent mean ± SD from two biological replicates (*n* = 6 technical replicates). All significance levels are denoted as *P ≤ 0.05, **P ≤ 0.01, ***P ≤ 0.001, ****P ≤ 0.0001. Analyses were performed using Mann-Whitney *U* tests (**A, B, G–J, L**) or two-way ANOVA with Šídák’s multiple comparisons test (**C–F**). The icons in panels E, F, I, J, and L were created in BioRender. Huang, D. (2025) https://BioRender.com/t8i3afr.

The DB-deficient DENV-2 (D2Y98P/ΔDB1-2), lacking both the DB1 and DB2 structure in domain II, was constructed based on the infectious clone of the wild-type (WT) DENV-2 strain D2Y98P (Fig.S1A and S1B). The length of the 3′UTR for WT was 454 nt, while it was reduced to 285 nt for D2Y98P/ΔDB1-2 (Fig. S1C). Sequencing confirmed the absence of the DB structure in D2Y98P/ΔDB1-2, with no mutations detected in the remaining region (Fig. S1D). Plaque assays in BHK-21 cells demonstrated severely attenuated plaque formation by D2Y98P/ΔDB1-2 compared to WT (no visible plaques vs 0.51 mm² average plaque area) (Fig. 1A). Consistently, immunostaining focus assays revealed significantly smaller infection foci for D2Y98P/ΔDB1-2 (0.36 mm² vs 0.98 mm² for WT; *P*_foci area_ < 0.0001) (Fig. 1B). These results underscored the importance of the DB structure in enhancing the plaque-forming ability of DENV-2.

### The DB structure enhances cytopathic effects and viral replication in mosquito cell lines

In C6/36 cells, D2Y98P/ΔDB1-2 exhibited significantly higher cell viability than WT (*P* < 0.000001) (Fig. 1G), with multiplicity of infection (MOI) dependent enhancement (Fig. S2A and S2E), whereas no viability changes were observed in Aag2 cells (Fig. 1H; Fig. S2B and S2F). Additionally, D2Y98P/ΔDB1-2 did not induce cytopathic effects (CPE) in either C6/36 or Aag2, unlike the WT strain, which caused notable vacuolation in C6/36 cells post-infection (Fig. S3).

D2Y98P/ΔDB1-2 exhibited significantly reduced viral loads compared to WT. In C6/36 cells, D2Y98P/ΔDB1-2 was consistently 5- to 15-fold lower than that of the WT (peak 10^8.4^ copies/µL vs 10^9.1^ copies/µL for WT) (Fig. 1C). In Aag2 cells, D2Y98P/ΔDB1-2 showed significantly lower viral genome levels in the supernatant, with a maximum of 10^5.4^ copies/µL at 7 d.p.i. (Fig. 1D). The WT ranged from 10^4.2^ to 10^7.6^ copies/µL (Fig. 1D). The D2Y98P/ΔDB1-2 displayed reduced viral replication capacity in mosquito cell lines compared to the WT and showed a weaker replication capacity in Aag2 cells, where the immune response was intact.

These findings suggested that the presence of a functional DB structure was essential for efficient viral replication in mosquito cell lines and may contribute to the modulation of antiviral immune responses.

### The DB structure enhances cytopathic effects without impairing viral replication in vertebrate cell lines

In tested vertebrate cell lines, the D2Y98P/ΔDB1-2 consistently demonstrated higher cell viability than WT (*P* < 0.000001), especially for BHK-21 cells, which showed a viability of 61% and 32% after being infected with WT at 5 and 7 d.p.i., respectively (Fig. 1I). The D2Y98P/ΔDB1-2 infection in Vero E6 cells resulted in a 99% viability rate, surpassing the 71% seen with WT (*P* < 0.000001) (Fig. 1J). At MOI = 1, D2Y98P/ΔDB1-2 maintained higher cell viability than WT (*P* < 0.0001; Fig. S2C, S2D, S2G and S2H). Notably, D2Y98P/ΔDB1-2-infected BHK-21 and Vero E6 cells showed no CPE, whereas WT caused cell rounding and detachment (Fig. S3).

Viral load analysis revealed that D2Y98P/ΔDB1-2 reached peak levels in the supernatant at 4 d.p.i., with a subsequent trend paralleling that of WT from 5 to 7 d.p.i. (Fig. 1E and F). In BHK-21 and Vero E6, D2Y98P/ΔDB1-2 viral genome copy numbers peaked at 10^8.2^ and 10^7.1^ copies/μL, respectively, similar to WT levels of 10^8.0^ and 10^7.8^ copies/μL (Fig. 1E and F).

The data indicated that the DB structure enhanced cellular death following viral infection, without affecting viral replication efficiency in vertebrate cell lines.

### The DB structure affects sfRNA production and increases accumulation

Studies have also shown that the 3′UTR structure influenced the production of sfRNAs. Northern blot analysis was used to compare sfRNAs in various cell lines infected with D2Y98P/ΔDB1-2 and WT (Fig. 1K). WT mainly produces two types of sfRNAs, sfRNA1 and sfRNA2 (423 nt and 348 nt, respectively), resulting from Xrn1 stalling at SLI and SLII (Fig. 1K; Fig. S1A). D2Y98P/ΔDB1-2 primarily produces one type of sfRNA, sfRNA1’ (255 nt), derived from Xrn1 stalling at SLI (Fig. 1K; Fig. S1B). When lacking DB structures, D2Y98P/ΔDB1-2 produced shorter sfRNA of 255 nt compared with 423 nt for WT, which generated longer sfRNA. The sfRNA phenotypes of D2Y98P/ΔDB1-2 and WT were consistent in both mosquito and vertebrate cell lines (Fig. 1K). qRT-PCR was used to measure the ratio of sfRNAs to genomic RNA (Fig. 1L). Mosquito cell lines notably exhibited a higher abundance of sfRNAs compared to mammalian cell lines for both viral strains. These findings suggested that the DB structure regulated sfRNA production and accumulation, potentially playing a critical role in DENV-2 infection within mosquitoes.

### DB structure is conducive to DENV infection in ***Ae. aegypti*** **and** ***Ae. albopictus*** **mosquito**

**Fig 2:**
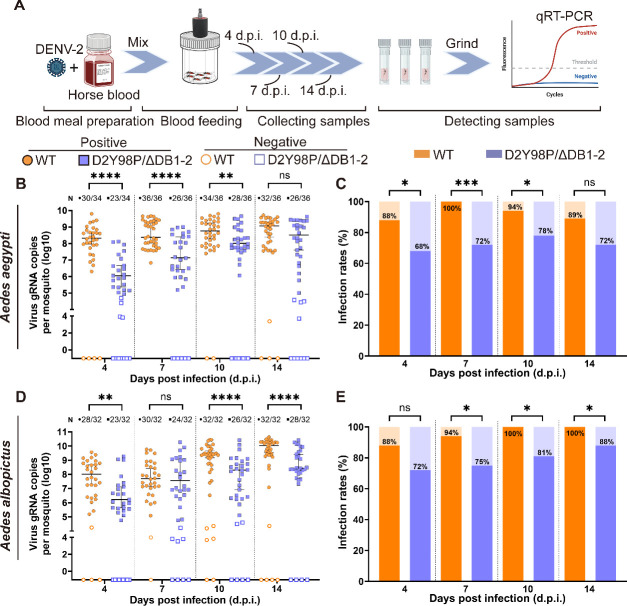
The effects of DB structure on DENV-2 infection in *Ae. aegypti* and *Ae. albopictus*. (**A**) The diagram illustrates the process of oral infection. Adult female mosquitoes were fed on blood meal containing 10^6.5^ PFU∙mL^−1^ of DENV-2. Engorged mosquitoes were collected and harvested at 4, 7, 10, and 14 d.p.i. for viral RNA quantification. (**B**) Viral RNA copies and (**C**) infection rates were measured after *Ae. aegypti* infection; (**D**) viral RNA copies and (**E**) infection rates were measured after *Ae. albopictus* infection. For the scatter dot plot (**B and D**), each data point represents an individual mosquito. N: detected positive mosquito numbers/detected mosquito numbers. Bar is expressed as the mean with 95% CI. Viral RNA copies analysis was performed using Welch’s *t* test. Differences in the rates were analyzed with chi-square test. All significance levels are denoted as *P ≤ 0.05, **P ≤ 0.01, ***P ≤ 0.001, ****P ≤ 0.0001. Panel A was created in BioRender. Huang, D. (2025) https://BioRender.com/pg9dkfy.

To determine the impact of DB structure in mosquito infection of DENV-2, both female *Ae. aegypti* and *Ae. albopictus* mosquitoes were exposed to D2Y98P/ΔDB1-2 and WT through blood feeding with 10^6.5^ PFU∙mL^−1^ viruses (Fig. 2A). Viral RNA copies were then quantified for each exposed single mosquito at 4, 7, 10, and 14 d.p.i. to assess viral replication and infection rate (IR).

In *Ae. aegypti*, D2Y98P/ΔDB1-2 was detectable in all exposed mosquitoes with IR recorded as 68%, 72%, 78%, and 72% at the respective time points (Fig. 2C). The IR for D2Y98P/ΔDB1-2 at 7 d.p.i. was significantly lower (*P* = 0.0007) than WT where IR was 100%. Viral RNA copies number in D2Y98P/ΔDB1-2-infected mosquitoes increased over time from 10^8.3^ to 10^9.1^ copies/mosquito (Fig. 2B). However, these levels were significantly lower than those in WT-infected mosquitoes at 4, 7, 10, and 14 d.p.i. (*P*_4 d.p.i_*_._* < 0.0001, *P*_7 d.p.i_*_._* < 0.0001, *P*_10 d.p.i_*_._* = 0.0087, *P*_14 d.p.i_*_._* = 0.0479), with a 35-fold difference at 4 d.p.i. (Fig. 2B).

For *Ae. albopictus*, D2Y98P/ΔDB1-2 viral loads were significantly lower than WT at 4, 10, and 14 d.p.i. (*P*_4 d.p.i_*_._* = 0.0013, *P*_10 d.p.i_*_._* = 0.0002, *P*_14 d.p.i_*_._* < 0.0001) (Fig. 2D). The IR of D2Y98P/ΔDB1-2 was lower than that of the WT group at all detection time points (Fig. 2E). At 7, 10, and 14 d.p.i., the IR of D2Y98P/ΔDB1-2 (IR_7 d.p.i._ = 75%, IR_10 d.p.i._ = 81%, IR_14 d.p.i._ = 88%) in *Ae. albopictus* was significantly lower (*P*_7 d.p.i._ = 0.0389, *P*_10 d.p.i_*_._* = 0.0101, *P*_14 d.p.i_*_._* = 0.0389) than WT where IR was 94%, 100%, and 100%, respectively (Fig. 2E).

This result showed that DB structure enhanced DENV-2 infection efficiency in *Aedes* mosquitoes because D2Y98P/ΔDB1-2 showed significantly lower infection rates and viral loads compared to WT in both *Ae. aegypti* and *Ae. albopictus* mosquitoes.

Lesions were also observed in the midgut of *Ae. aegypti* mosquitoes infected through blood feeding at 7 d.p.i. (Fig. S5). Compared to uninfected midgut tissue, D2Y98P/ΔDB1-2 infection led to denser midgut tissue with fewer endoplasmic reticulum vacuoles and increased autophagosomes (Fig. S5B and S5E), whereas WT infection caused midgut tissue loosening with numerous vacuoles and ribosome aggregations (Fig. S5A). The presence of mature virus particles was more pronounced in WT-infected midguts (Fig. S5D), suggesting that the DB structure may strengthen the viral pathogenicity to the midgut of mosquitoes.

### DB structure is required for DENV-2 to escape midgut infection barrier and midgut escape barrier

**Fig 3:**
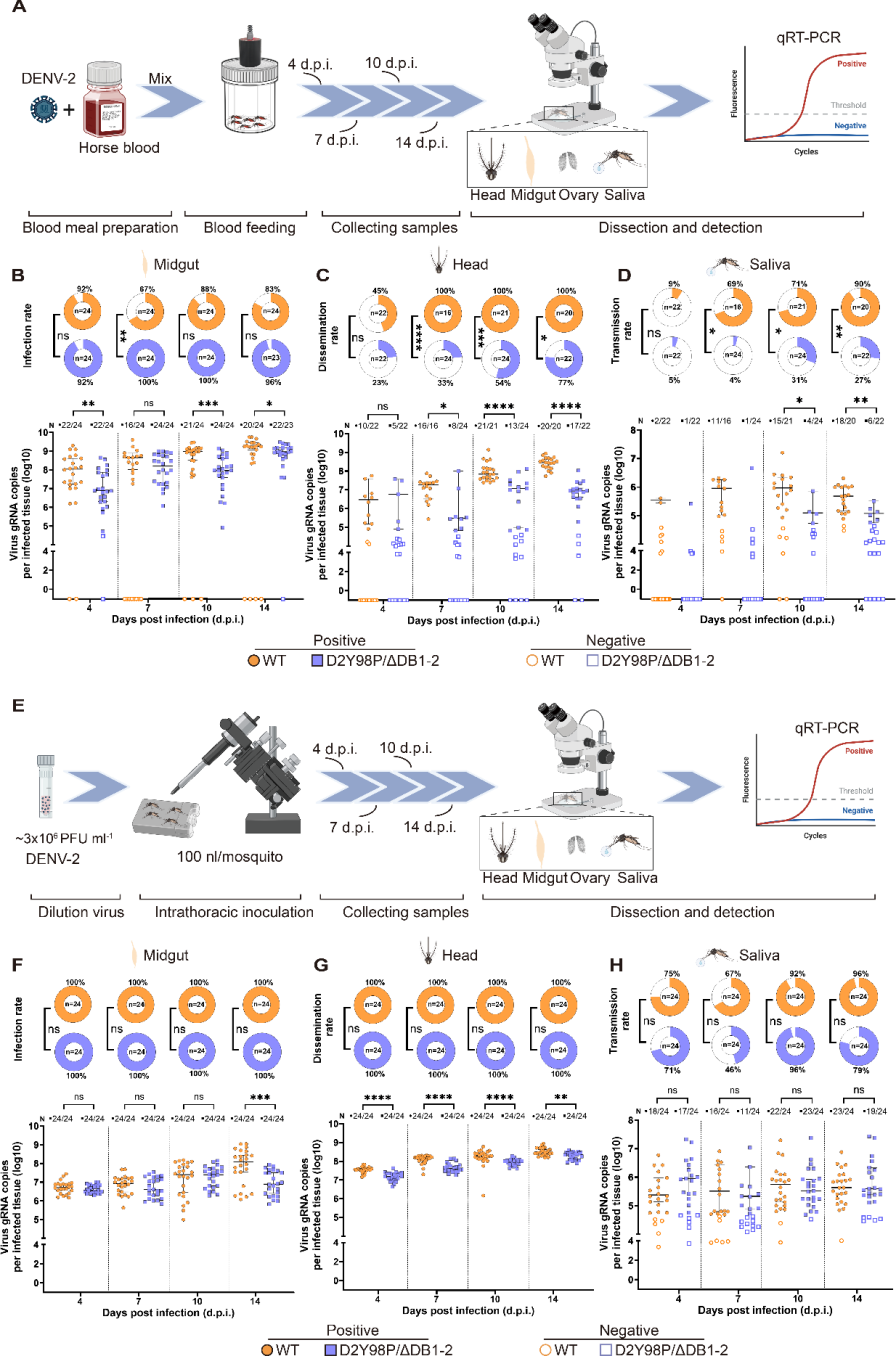
DENV-2 infection in different tissues of *Ae. aegypti* through oral infection or intrathoracic inoculation. (**A, E**) Schematic of a flow chart for the detection of infection in different tissues of *Ae. aegypti* through (**A**) oral infection or (**E**) intrathoracic inoculation. (**B–D and F–H**) Infection results of *Ae. aegypti* in different tissues and saliva. The mosquito’s (**B, F**) midgut, (**C, G**) head, and (**D, H**) saliva were tested by qRT-PCR at 4, 7, 10, and 14 d.p.i.. For scatter dot plot, each dot represents a tissue or saliva sample. N: detected positive tissue (saliva sample) number/detected tissue (saliva sample) numbers. Bar is expressed as the mean with 95% CI. Viral RNA copies analysis was performed using Welch’s *t* test. The circular plot indicates the infection rates of the midgut, head, and saliva, respectively. Differences in the rates were analyzed with the chi-square test. All significance levels are denoted as *P ≤ 0.05, **P ≤ 0.01, ***P ≤ 0.001, ****P ≤ 0.0001. Panels A and E were created in BioRender. Huang, D. (2025) https://BioRender.com/pg9dkfy.

To determine the role of DB structure in the mosquito transmission of DENV-2, female *Ae. aegypti* were exposed to WT and D2Y98P/ΔDB1-2 by blood feeding or intrathoracic (i.t.) injection (Fig. 3A and E). Viral RNA copies in midgut, head, and saliva at 4, 7, 10, and 14 d.p.i. were measured.

In the midgut, the infection rate increased with the days of infection by blood feeding. Infection rate of D2Y98P/ΔDB1-2 infected mosquitoes was higher (IR_4 d.p.i._: 92%, IR_7 d.p.i._: 100%, IR_10 d.p.i._:100%, IR_14 d.p.i._: 96%) than WT, with the average viral RNA copies escalating from 10^6.9^ to 10^9.0^ copies (Fig. 3B). The mean virus copies of D2Y98P/ΔDB1-2 were significantly lower than that of WT at 4, 7, 10, and 14 d.p.i. (*P*_4 d.p.i_*_._* = 0.0035, *P*_7 d.p.i_*_._* = 0.0431, *P*_10 d.p.i_*_._* = 0.0003, *P*_14 d.p.i_*_._* = 0.0352). When mosquitoes were infected using i.t. injection, viruses bypassed the MIB and MEB, ensuring that every mosquito was infected. The viral load in D2Y98P/ΔDB1-2 was significantly lower than WT only at 14 d.p.i. (*P*_14 d.p.i_*_._* = 0.0007) (Fig. 3F). The results showed that the DB structure was beneficial to viral replication and enabled the virus to escape MIB, as the virus’s ability to reproduce was weakened when the DB structure was absent, but it could still penetrate the MIB.

In the head, D2Y98P/ΔDB1-2 viral loads were significantly lower than WT at 7, 10, and 14 d.p.i. (*P*_7 d.p.i._ = 0.0202, *P*_10 d.p.i._ = 0.0022, *P*_14 d.p.i._ ≤ 0.0001) (Fig. 3C). At 14 d.p.i., the average viral load in positive heads was only 10^7.0^ copies for D2Y98P/ΔDB1-2, but 10^8.4^ copies for WT representing a 30-fold increase compared to D2Y98P/ΔDB1-2. The D2Y98P/ΔDB1-2 showed a significantly lower dissemination rates (DR) compared to WT of 33% at 7, 54% at 10, and 77% at 14 d.p.i. (*P*_7 d.p.i_*_._* < 0.0001, *P*_10 d.p.i._ = 0.0004, *P*_14 d.p.i._ = 0.0231) (Fig. 3C). After inoculation via i.t. injection, D2Y98P/ΔDB1-2 maintained a 100% DR, but with significantly lower viral RNA copies than WT (*P*_4 d.p.i_*_._* < 0.0001, *P*_7 d.p.i_*_._* < 0.0001, *P*_10 d.p.i_*_._* = 0.0147, *P*_14 d.p.i._ = 0.0005) (Fig. 3G). The results showed that the deficiency of DB structure weakened the ability of the virus to penetrate the MEB. The data suggested that the DB structure could facilitate viral penetration through both MIB and MEB. Once the virus bypassed both MIB and MEB, it rapidly disseminated throughout the mosquito’s hemolymph, reaching all parts of its body.

### DB structure is not essential for DENV-2 to escape the salivary gland infection and salivary gland escape barriers

The virus will eventually reach the salivary gland, penetrate the SGIB, infect the gland, and penetrate the SGEB to enter saliva and spread to the next host through mosquito bites. Following blood feeding, D2Y98P/ΔDB1-2 showed significantly reduced transmission rates (TR) and viral copy numbers in saliva compared to WT, with TRs of 5%, 4%, 31%, and 27% at 7, 10, and 14 d.p.i. (*P*_7 d.p.i._ < 0.0001, *P*_10 d.p.i._ = 0.0002, *P*_14 d.p.i._ < 0.0001), compared with 9% at 4 d.p.i. rising to 90% at 14 d.p.i. for WT (Fig. 3D).

It was notable that D2Y98P/ΔDB1-2’s mean viral load was lower than WT at 10 (*P*_10 d.p.i._ = 0.0417) and 14 d.p.i. (*P*_14 d.p.i._ = 0.0033) (Fig. 3D). In contrast, after i.t. injection, TR for D2Y98P/ΔDB1-2 increased but did not differ significantly from WT at 71% at 4 d.p.i., 46% at 7 d.p.i., 96% at 10 d.p.i., and 79% at 14 d.p.i. (Fig. 3H). These findings suggested that the virus was capable of effectively infecting the salivary glands by bypassing the midgut barrier, penetrating both SGIB and SGEB and subsequently secreting into saliva. This implied that the DB structure was crucial for midgut rather than salivary gland infection.

The collective findings indicated that D2Y98P/ΔDB1-2 could establish midgut infection and overcome both the MIB and MEB, as evidenced by the detection of viral RNA in the head and saliva, but D2Y98P/ΔDB1-2’s replication, dissemination, and transmission were significantly less efficient than that of WT. The predominant localization of D2Y98P/ΔDB1-2 in the midgut of *Ae. aegypti* underscored the DB structure’s importance in facilitating productive infection and efficient viral transmission.

### DB structure may facilitate virus escape by modulating immune the effector DefC

**Fig 4:**
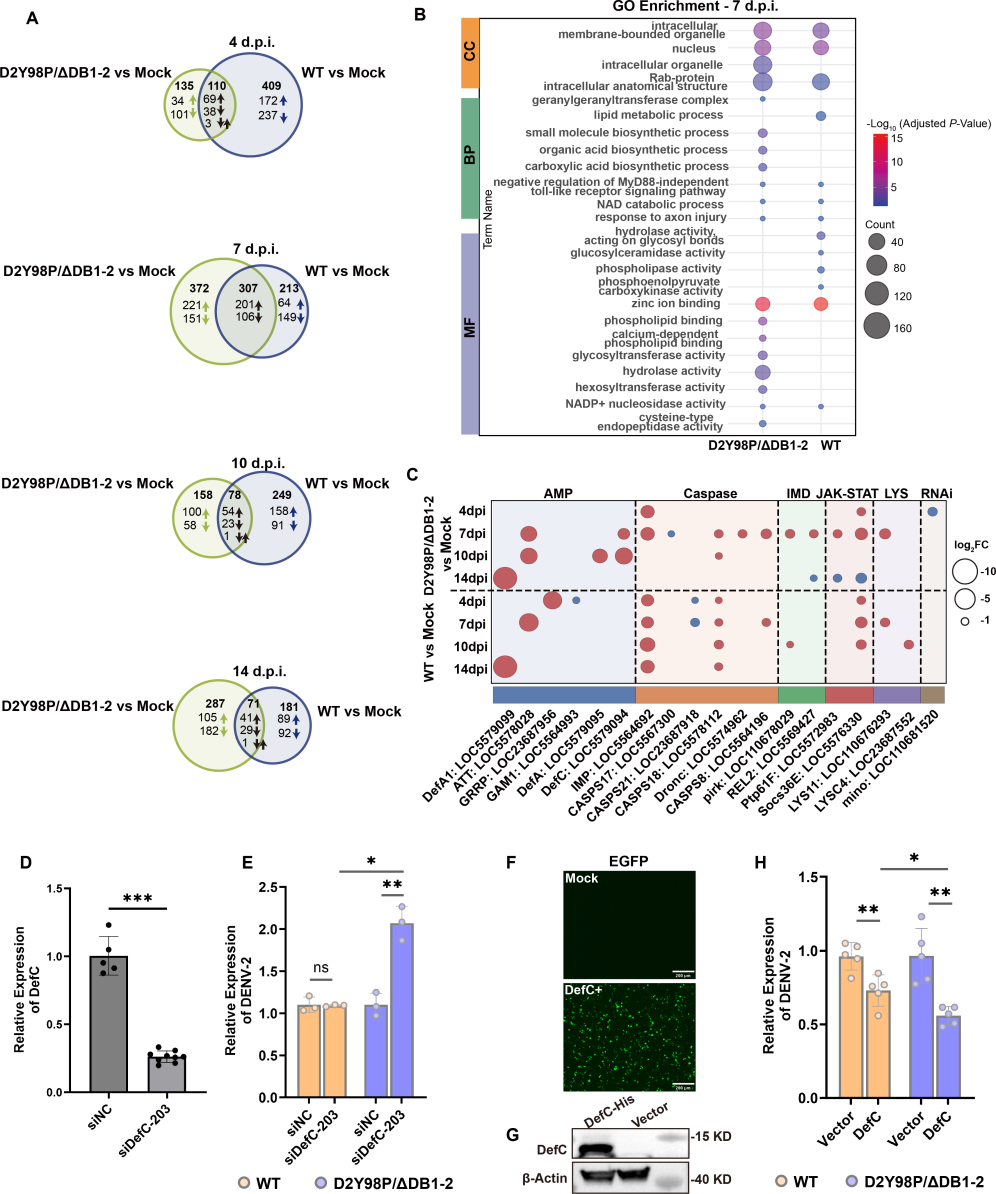
Differential gene expression analysis and verification in *Ae. aegypti* and mosquito cells infected by WT and D2Y98P/ΔDB1-2. (**A**) Venn diagram showing the overlap between differentially expressed genes (DEGs) from D2Y98P/ΔDB1-2 and WT-infected *Ae. aegypti* at different times. (**B**) The bubble plot illustrates enriched Gene Ontology (GO) categories associated with DEGs at 7 d.p.i. CC, cellular components; BP, biological processes; MF, molecular functions. The bubble color represents −Log (adjusted *P*-value), and the size represents gene count. (**C**) The bubble plot displays the expression of immune genes following infection with D2Y98P/ΔDB1-2 or WT in *Ae. Aegypti* at different time points. Red represents gene upregulation, blue represents gene downregulation, and the size of bubble represents log_2_ (fold change). DEGs were considered significant if adjusted *P*-values < 0.05. (**D**) The knockdown efficiency of DefC in C6/36 cell lines was measured by qRT-PCR. Data are expressed as the mean with 95% CI from three biological replicates (*n* = 5 technical replicates). (**E**) DENV-2 replication in C6/36 cell lines after DefC knockdown. Viral replication was detected by qRT-PCR. WT is represented by orange and D2Y98P/ΔDB1-2 by purple. Data are presented as the mean ± SD from three biological replicates (*n* = 5 technical replicates). (**F**) Fluorescence detection for plasmid overexpression. EGFP represents enhanced green fluorescent protein. The observation of green fluorescence indicates that the plasmid has been successfully transfected into C6/36 cell lines. (**G**) C6/36 cells were treated with overexpression plasmid (pAC5.1-DefC) targeting DefC, and the overexpression was confirmed by western blot analysis. (**H**) DENV-2 replication in C6/36 cell lines after DefC overexpression. Virial replication detected by qRT-PCR in WT (orange) or D2Y98P/ΔDB1-2 (purple). Bars represent mean ± SD from three biological replicates (*n* = 5 technical replicates). All significance levels are denoted as *P ≤ 0.05, **P ≤ 0.01, ***P ≤ 0.001, ****P ≤ 0.0001. Analyses were performed using Mann-Whitney *U* tests (**D**) or Welch’s *t* test (**E and H**).

To investigate how the DB structure affected DENV-2 escape in *Ae. aegypti* mosquitoes, the midgut transcriptome at 4, 7, 10, and 14 d.p.i. of WT and D2Y98P/ΔDB1-2 was profiled. After D2Y98P/ΔDB1-2 infection, the highest number of differentially expressed genes (DEGs) was observed at 7 d.p.i. including 679 DEGs, with 422 genes upregulated and 257 genes downregulated (Fig. 4A). In conjunction with mosquito infection experiments (Fig. 3B), it suggests that the virus undergoes robust replication, triggering the *Ae. aegypti* midgut’s immune response and exerting an impact on the physiological function of the host.

Immune-related gene expression analysis found that only the IMP and Socs36E genes exhibited upregulation at 4 d.p.i. following D2Y98P/ΔDB1-2 infection (Fig. 4C) when the virus established replication in the midgut and only a limited number of viruses successfully penetrating the MEB (Fig. 3B and C). At 7 d.p.i., extensive replication of D2Y98P/ΔDB1-2 occurred in the midgut (Fig. 3B). The Rel-like transcription factor 2 (REL2) and two antimicrobial peptides (AMPs) DefC and attacin (ATT) were significantly upregulated to hinder viral escape from the MEB (Fig. 3C and 4C). At 10 d.p.i., approximately half of D2Y98P/ΔDB1-2 had successfully evaded the MEB as it spread into hemolymph toward the head and salivary glands (Fig. 3C and D). During this period, only AMPs including defensin A (DefA), DefC, and ATT and caspase genes exhibited upregulation in the midgut to inhibit D2Y98P/ΔDB1-2 dissemination. At 14 d.p.i., a substantial number of viruses had disseminated from the midgut to other tissues in the D2Y98P/ΔDB1-2 or WT during infections (Fig. 3B and C), possibly reaching an equilibrium state between virus and host where the host’s immune response is regulated at a lower level to sustain virus persistence and transmission (Fig. 4C).

It was notable that at 10 d.p.i. for DefA (log_2_FC = 3.36) and 7 and 10 d.p.i. for DefC (log_2_FC = 2.23 and log_2_FC = 4.56, respectively), they were significantly upregulated specifically when D2Y98P/ΔDB1-2 was actively escaping the midgut barrier in large numbers. Previous work by Pan et al. found that DefA and DefC inhibited the titer of DENV-2 in *Ae. aegypti* mosquitoes (26). Based on our data, DefA and DefC are only upregulated during D2Y98P/ΔDB1-2 infection, but not in WT, so they may be acting as limiting factors affecting viral replication and may hinder virus escape from the midgut epithelial barrier.

Several genes, namely, DefA, DefC, REL2, and Protein tyrosine phosphatase 61F (Ptp61F), which were upregulated only during the infection process of D2Y98P/ΔDB1-2, were, respectively, knocked down in C6/36 cells. Among them, only the knockdown efficiencies of siDefC-203 and siREL2-547 exceeded 70%, which were 74% and 76%, respectively (Fig. 4D; Fig. S6). Subsequently, viruses were inoculated to evaluate viral RNA replication levels. The knockdown of REL2 had no significant effect on viral replication (Fig. S6). However, after DefC knockdown, D2Y98P/ΔDB1-2 replication exhibited a significant increase compared to both the control group and the WT group (*P*_D2Y98P/ΔDB1-2 vs NC_ = 0.0035, *P*_D2Y98P/ΔDB1-2 vs WT_ = 0.0134) (Fig. 4E). We further overexpressed the DefC gene (Fig. 4F and G). Upon overexpression, the replication levels of both D2Y98P/ΔDB1-2 and WT strains decreased significantly (*P*_D2Y98P/ΔDB1-2 vs NC_ = 0.0062, *P*_WT vs NC_ = 0.0065) (Fig. 4H). Specifically, the decline in D2Y98P/ΔDB1-2 replication was more pronounced than that in the WT strain (*P*_D2Y98P/ΔDB1-2 vs WT_ = 0.0196) (Fig. 4H).

These results indicated that DB structure had an immune escape function, with significant activation of AMPs, IMD, and JAK-STAT including ATT, DefC, DefA, REL2, and Ptp61F. Among them, the replication of D2Y98P/ΔDB1-2 was upregulated after DefC knockdown, and the replication of D2Y98P/ΔDB1-2 was decreased after DefC overexpression, indicating that the DB structure may promote the escape of the virus from the midgut barrier by regulating the expression of DefC.

## DISCUSSION

The synergistic pressures of climate change and globalization have amplified DENV spread, necessitating deeper insights into its transmission mechanisms. While previous studies established that flaviviral DB structures regulate genome replication in human and mosquito cells (4, 19), our findings demonstrate their critical role in *Aedes aegypti* midgut evasion and immune modulation—a pivotal advance in understanding DENV transmission dynamics.

We found that the absence of both of the DB structures in D2Y98P/ΔDB1-2 significantly attenuated DENV-2 replication in mosquito cell lines and reduced CPE (Fig. 1C through J), suggesting a pivotal role in mosquito infection and transmission. This attenuation was not limited to the mosquito cells, as D2Y98P/ΔDB1-2 also showed reduced replication in mammalian cells, indicating a broader impact on viral fitness.

Based on these findings, the effect of the DB structures on DENV-2 infection and transmission in *Ae. aegypti* was further investigated. There is evidence in the literature suggesting that the SL structure was a critical factor in facilitating the penetration of both WNV and ZIKV through the salivary gland barrier, promoting efficient virus transmission (20–22). Our study made a novel discovery that the DB structures predominantly facilitated viral evasion of the midgut barrier rather than the salivary gland barrier during DENV-2 infection in *Ae. aegypti*, as the absence of a DB structure enabled the virus to primarily localize in the midgut. While the virus effectively penetrated MIB in mosquitoes, it displayed diminished ability to breach the MEB, thereby affecting its transmission potential (Fig. 3B through 3D). This observation was further supported by intrathoracic injection data that circumvented the midgut barrier (Fig. 3F through 3H). Interestingly, the DB structures did not appear to be the key factor in the virus’s ability to break through the salivary gland barrier, whether it involved the SGIB or the SGEB.

When a mosquito is infected by the virus, it initially triggers the innate immune response of the mosquito to combat the viral infection. The midgut transcriptome analysis revealed that DefA, DefC, REL2, and Ptp61F were significantly upregulated at 7 and 10 d.p.i. in D2Y98P/ΔDB1-2, whereas no upregulation was observed in the WT (Fig. 4C). We further verified that DefC has an inhibitory effect on the replication of D2Y98P/ΔDB1-2 through knockdown and overexpression. Compared with WT, the inhibitory effect on D2Y98P/ΔDB1-2 is more obvious. Although previous studies have shown that DefC, as an important antimicrobial peptide, plays a significant role in mosquitoes’ resistance to viral infection and transmission (26), our findings have further focused on the DB structures in DENV-2, discovering DB structures may be the key structure for the virus to regulate immune genes in mosquito hosts to facilitate virus escape from MEB. It also noted that the negative regulatory factors Ptp61F and Socs36E are upregulated to inhibit the JAK-STAT pathway to facilitate viral replication (27). The virus and host establish a balance through the activation and inhibition of immune pathways, maintaining the persistent replication and transmission of the virus.

Understanding the molecular mechanism by which the DB structures regulate immune gene expression will help to develop new intervention strategies to suppress virus infection or transmission in mosquitoes. This study exclusively focused on the reported immune genes, leaving a vast number of unknown mosquito genes unexplored. Regarding DEGs identified solely after D2Y98P/ΔDB1-2 infection (Table. S2), their role in viral evasion remains ambiguous and necessitates further investigation. In addition, the DefC gene has only been verified in cell lines so far. Subsequently, knock-down and overexpression experiments can be conducted in mosquitoes to further verify the results.

During flavivirus infection, sfRNAs are produced, which, as functional subgenomic RNAs, can inhibit RNAi and the Toll pathway within mosquitoes (20, 23). These sfRNAs have been detected in the saliva of mosquitoes infected with DENV, enhancing viral transmissibility (28), with sfRNA1 playing a role in facilitating the dissemination of WNV in the midgut (20, 29). The above studies have shown that sfRNAs may regulate virus infection in the mosquito host and that the absence of the DB structures in D2Y98P/ΔDB1-2 resulted in a shorter sfRNA (Fig. 1F), which may be one of the factors limiting its ability to breach the midgut barrier and spread effectively. However, the molecular mechanisms underlying sfRNA regulation in the midgut are not yet clear and warrant further investigation.

The DB structure in DENV exhibits relatively conserved sequences, but distinct evolutionary models can be observed for secondary structure between DB1 and DB2 across different serotypes. The DB secondary structures among different serotypes are evolutionarily higher than those within the same serotype (4). This suggests that DB structures of different serotypes have divergent evolutionary pathways (4, 30). Further investigations are required to explore the impact of the DB structure on other DENVs. The 3′UTR of DENV often shows genetic changes that aid viral circulation and adaptation to different hosts (30, 31), highlighting the need for further investigation into the impact of DB structure on cross-species transmission. The exploration of the DB structure’s role in other flaviviruses and its potential as a target for broad-spectrum anti-arboviral therapies also warrants further investigation.

Deletions of DB structures have emerged as a promising strategy for developing attenuated vaccines against flaviviruses. Specifically, the deletion of the 30 nt sequence at DB2 has been successfully attenuated DENV-1 and DENV-4, while a combined deletion of the 31 nt sequence at DB1 and the 30 nt sequence at DB2 has been used to attenuate DENV-3 (32–34). However, these strategies have proven ineffective for DENV-2, highlighting the unique challenges associated with this serotype (35, 36). In our study, we found that deleting both DB1 and DB2 on DENV-2 significantly reduced the cytopathogenicity of DENV-2 in both vertebrate and mosquito cell lines. But our findings on viral replication capacity differ from those reported by Luana de Borba et al. in mammalian cell lines (24). This discrepancy likely stems from the differences in the cell models used. Their study used IFN-competent A549 cells, while ours employed IFN-deficient BHK-21 and Vero E6 cells. In A549 cells, WT and ΔDB1-2 showed significant replication differences, possibly due to sfRNA-mediated IFN inhibition (37, 38). In contrast, in BHK-21 and Vero E6 cells with weak IFN responses, sfRNA defects have minimal effects on viral replication. It is speculated that sfRNA1’ has a weaker IFN-inhibitory effect than sfRNA1 and sfRNA2. Moreover, while previous vaccine candidates showed restricted replication and transmission in *Aedes* mosquitoes (36, 39–41), our virus still retained a 27% transmission rate in *Ae. aegypti*, which is higher than previously reported. This is a limitation for vaccine candidate use. We can apply this strategy to virus strains with lower transmissibility or further optimize the deletion approach to identify vaccine candidates with good immunogenicity and high safety. Future work should focus on evaluating the immunogenicity and protective efficacy of D2Y98P/ΔDB1-2 in vertebrate models to validate their potential as vaccine candidates. This approach may provide a novel strategy for developing a universal flavivirus vaccine.

In conclusion, this study provides insights into the complex interactions between DENV-2 and its mosquito vector, emphasizing the role of the viral DB structure in modulating DefC expression to facilitate viral escape. These findings have implications for understanding the molecular mechanisms underlying DENV transmission and may inform strategies for the control of dengue fever in endemic regions.

## MATERIALS AND METHODS

### Cell culture

Baby hamster kidney cells (BHK-21; RRID: CVCL_1914) and African green monkey kidney cells (Vero E6; RRID: CVCL_0574) were cultured in Dulbecco’s modified Eagle’s medium (DMEM; Gibco, USA) supplemented with 10% (vol/vol) fetal bovine serum (FBS; Morgate, AUS), 100 units/mL of penicillin and 100 µg/mL of streptomycin. All mammalian cells were grown at 37°C under a 5% CO_2_ atmosphere. The *Ae. albopictus* mosquito cell line (C6/36; RRID: CVCL_Z230) was cultivated in Roswell Park Memorial Institute 1640 medium (RPMI; Gibco, USA) supplemented with 10% (vol/vol) FBS, 100 units/mL penicillin, and 100 µg/mL streptomycin. The *Ae. aegypti* mosquito cell line (Aag2; RRID: CVCL_C3S1) was cultured in Schneider’s Drosophila medium (SDM; Gibco, USA) containing 10% (vol/vol) FBS, 100 units/mL penicillin, and 100 µg/mL streptomycin. Insect cells (C6/36 and Aag2) were cultured at 28°C in an incubator with 5% CO_2_.

### Viruses

DENV-2 virus strain (D2Y98P) and DB-deficient of DENV-2 virus strain (D2Y98P/ΔDB1-2) were rescued from full-length cDNA clones (pACYC-D2Y98PPFL and pACYC-D2Y98P/ΔDB1-2). Viral RNA was transcribed from full-length cDNA clones *in vitro*, following the protocol provided by mMESSAGE mMACHINE Kit (ThermoFisher, USA). Viral stocks were obtained by electroporation of 10 µg of *in vitro* transcribed viral RNA into BHK-21 and propagated for one passage (P1) in Vero E6 cells, following previously established protocols (42, 43). Supernatants were harvested at 7 days post-infection (d.p.i.), and viruses were quantified by plaque assays or immunostaining focus assays. All work with infectious viruses was conducted in a biosafety level-2 (BSL-2) laboratory.

### Construction of recombinant DENV-2

Mutations were introduced into the full-length cDNA of DENV-2 (pACYC-D2Y98PPFL, strain D2Y98P), replacing the NruI-ClaI fragment of the WT plasmid with a fragment derived from overlapping PCRs containing the desired mutation as previously described (24, 25). The resulting constructs were cultured in *Escherichia coli* TOP 10 strain (Sangon Biotech, CHN) and subjected to sequencing for validation of the mutations (Sangon Biotech, CHN). All restriction enzymes and DNA polymerases utilized in this study were procured from New England Biolabs (NEB, USA) and Takara Bio (Takara, JPN), respectively.

### Plaque assay and immunostaining focus assay

D2Y98P titers were determined using a plaque assay method. Serial dilutions of the cell culture supernatant were prepared in DMEM media (containing 2% FBS, 100 units/mL penicillin, and 100 µg/mL streptomycin). Each dilution (100 µL) was used to infect BHK-21 cells grown in 24-well plates with 1 × 10^5^ cells per well. After incubating with the inoculum for 1 h, an overlay media consisting of DMEM (containing 2% FBS, 100 units/mL penicillin, and 100 µg/mL streptomycin) mixed with 2% carboxymethyl cellulose (Sigma-Aldrich, USA) was added to the cells. The infected cells were then further incubated in a 37°C incubator with 5% CO_2_. At 4 d.p.i., cells were fixed with 500 µL/well of 4% formaldehyde for 30 min at room temperature. The formaldehyde was discarded and then stained with crystal violet staining solution (Beyotime, CHN) for 10 min. Finally, virus replication plaques were counted.

For immunostaining focus assay, the steps for infection were the same as plaque assays. At 4 d.p.i., cells were fixed with 500 µL/well of acetone and methanol (1:1) for 1 h at −20°C and washed with PBS three times and incubated with 150 µL/well of mouse monoclonal antibody to DENV-2 NS3 protein (GeneTex, USA) diluted in 1:1,000 for overnight at 4°C, followed by 1 h incubation with 150 µL/well of 1:500 dilution of HRP-conjugated goat anti-mouse secondary antibody (Proteintech, CHN). The Enhanced HRP-DAB Chromogenic kit (Tiangen, CHN) was used at room temperature for 10 min. All antibodies were diluted with PBS. After each incubation with antibody, plates were washed three times with PBS. Virus replication foci were counted, and titers were determined based on dilution factors.

### Mosquito rearing

*Ae. aegypti* (Rockefeller strain) were acquired from the Laboratory of Tropical Veterinary Medicine and Vector Biology at Hainan University. *Ae. albopictus* (Jiangsu strain) were from the Department of Vector Biology and Control, National Institute for Communicable Disease Control and Prevention, China CDC. *Ae. aegypti* and *Ae. albopictus* were reared from eggs and maintained as adults in a mosquito room under the following conditions: 28°C under light: dark cycle of 12:12 h and relative humidity of 75% ± 5%. Emerged adult mosquitoes were fed an 8% glucose solution until infection and maintained in mesh cages (30 × 30 × 30 cm) as previously described (44). Mosquitoes were reared in an arthropod containment level 1 (ACL-1) laboratory.

### Oral infection

For membrane-feeding infections, 5- to 8-day-old female mosquitoes were starved for 24 h. The mosquito infection experiments were conducted in an ACL-2 laboratory. Mosquitoes were fed with a mixture of defibrinated horse blood and virus supernatant containing 10^6^ PFU∙mL^−1^ of DENV-2, using an artificial feeding system (Hemotek, Lancashire, UK) covered with Hemotek feeding membrane. The mixed blood was quantified by plaque assays to measure viral titer. After blood feeding, mosquitoes were anesthetized using ice and immobilized on ice as previously described (44, 45). Fully engorged female mosquitoes were selected and placed into netting-covered containers with a capacity of 24 oz. Each group of 45 engorged female mosquitoes was then cultured in an incubator (28°C under light: dark cycle of 12:12 h and relative humidity of 75% ± 5%). Mosquito saliva, head, and midgut were collected at 4, 7, 10, and 14 d.p.i. for viral RNA determination using qRT-PCR as previously described (44, 46). The number of viral RNA copies was determined from a standard curve. All mosquitoes used in each experiment were hatched and raised under identical controlled conditions.

### Intrathoracic inoculation

The selected 5- to 8-day-old female mosquitoes were refrigerated and anesthetized before being placed on ice. The virus was injected into the mosquito thorax using a Nanoject III auto-nanoliter injector (Drummond Scientific, USA) under a microscope, with approximately 300 PFU at a volume of 100 nL per mosquito. The control group was injected with virus-free DMEM. Each group of 30 mosquitoes was then cultured in an incubator (28°C under light: dark cycle of 12:12 h and relative humidity of 75% ± 5%) and placed inside a container covered with netting as previously described (44, 46). Mosquito saliva, head, and midgut were collected at 4, 7, 10, and 14 d.p.i. for viral RNA determination using qRT-PCR. The number of viral RNA copies was determined from a standard curve. All mosquitoes used in each experiment were hatched and raised under identical controlled conditions.

### qRT-PCR

Total RNA extracted from individual mosquitoes or individual tissues by automated nucleic acid extraction system following the manufacturer’s instructions (NanoMagBio, CHN). RNA was using the CFX96 Touch Real-Time PCR Detection System (Bio-Rad) and HiScript II One Step qRT-PCR Probe Kit (Vazyme, CHN) to quantify viral RNA copies, following the manufacturer’s instructions. The specific primers are listed in Table S1.

### Standard curve

For DENV-2 viral copy number determination, viral RNA was extracted, and a specific region of the E gene was amplified using primers with T7 promoter sequence by RT-PCR (Takara, Japan). The PCR product was separated on a 1.2% agarose gel at 120V and running for 15 min, and using E.Z.N.A. Gel Extraction Kit (Omega, CHN) purified RT-PCR products. Subsequently, we used the T7 High Yield RNA Transcription kit (Vazyme, CHN) to transcribe RNA *in vitro* from the PT-PCR product containing the T7 promoter, following the instruction manual. The concentration of nucleic acid was measured using a Thermo Scientific NanoDrop One (Thermo Scientific, USA). To establish a standard curve for quantification, *in vitro* transcribed RNA was serially diluted 10-fold to obtain concentrations ranging from 10^0^ to 10^−10^. For the standard curves of gRNA and sfRNA, we referred to previous descriptions (47). Finally, we used qRT-PCR to detect viral RNA copies, with three technical replicates performed for each RNA sample. The specific primers and the equation for the standard curve are listed in Table S1.

### Viral growth kinetics

Mammalian cell lines BHK-21, Vero E6, or mosquito cell lines C6/36 and Aag2 were seeded in six-well plates at a density of 5 × 10^5^ and 1 × 10^6^ cells per well, respectively. Cells were infected with D2Y98P and D2Y98P/ΔDB1-2 at different MOIs (1, 0.1, and 0.01) for 1 h at 37℃ or 28℃. The inoculum was discarded, followed by washing with 1 × phosphate buffered saline (PBS; Gibco, USA) three times. The culture was then supplemented with fresh medium and placed in the incubator. The supernatant was harvested every day until 7 d.p.i. for quantification of viral genome copies, using qRT-PCR. For each cell line, two independent biological replicates were performed, with three technical replicates in each experiment.

### Cell viability

Mammalian cell lines BHK-21, Vero E6, or mosquito cell lines C6/36 and Aag2 were seeded in 96-well plates at a density of 1 × 10^4^ and 2 × 10^4^ cells per well, respectively. The D2Y98P and D2Y98P/ΔDB1-2 were diluted with the corresponding cell culture medium based on different MOIs (1, 0.1, and 0.01), and 100 µL of the virus diluent was added to each well. At the testing time point, 10 µL of CCK-8 solution (MedChemExpress, USA) was added to each well following the manufacturer’s instructions. The samples were then incubated in the incubator at 37℃ or 28℃ for 1 h and subsequently transferred to a refrigerator at 4℃ to terminate the reaction. Cell viability was quantified by measuring the absorbance at 450 nm on an Absorbance Microplate Reader (BioTek, USA). For each cell line, three independent biological replicates were performed, with six technical replicates in each experiment.

### Transmission electron microscopy

The midguts of 12 female mosquitoes infected through blood feeding at 7 d.p.i. were collected for analysis. Under a dissection microscope, the midguts were collected from the infected female mosquitoes. The collected tissues were then fixed in 2.5% glutaraldehyde to prepare them for further experimentation. Fixed samples were processed at the Center for Instrumental Analysis and Metrology (Wuhan Institute of Virology, China). The tissues were sectioned using an ultramicrotome and observed under a Tecnai G20 TWIN transmission electron microscope (FEI, USA). Image analysis and grouping were conducted using ImageJ and PowerPoint 2019.

### Northern blot

Mammalian cell lines BHK-21, Vero E6, or mosquito cell lines C6/36 and Aag2 were seeded in six-well plates at a density of 5 × 10^5^ and 1 × 10^6^ cells per well, respectively. The cells were infected with D2Y98P and D2Y98P/ΔDB1-2 at an MOI of 1 for 1 h in the incubator. After infection, the inoculum was removed, the cells were washed three times with 1× PBS, and fresh media was added. Samples were harvested at 72 h post-infection. At harvest, the media was aspirated, and cells were washed three times with 1× PBS. Total RNA was extracted from infected cells using Trizol (Takara, JPN), following the manufacturer’s protocol (48). For DENV genome and sfRNA detection, 15 ng of total RNA was separated on 5% polyacrylamide/7M urea gels (Solarbio, CHN) for 1 h at 180V, transferred onto a nylon membrane (Invitrogen, USA) using a 0.5× TBE transfer buffer (Coolaber, CHN) for 1 h at 380 mA, and crosslinked with UV radiation. The membrane was prehybridized at 37°C for 0.5 h in a prehybridization solution (Roche, USA), followed by overnight hybridization at 42°C in a hybridization solution (Roche, USA) containing biotin-labeled probes obtained and purified from Tsingke Biotech Company. The probe contained two RNA sequences targeting nucleotides 10664–10684 and 10700–10720 within the 3′UTR of DENV. Blots were subsequently washed twice with low stringency wash solution (Invitrogen, USA) at room temperature and then twice with high stringency wash solution (Invitrogen, USA) at 55°C. Finally, membranes were blocked using the Chemiluminescent Biotin-labeled Nucleic Acid Detection Kit (Beyotime, CHN), washed again for removal of excess reagents, and stained before visualization using a fully automatic chemiluminescence imaging system (Tanon, CHN). The sfRNA/gRNA ratio was determined based on the copy number, following the methodology previously described (47).

### RNA-seq and bioinformatic analysis

Total RNA was extracted from the midgut of mosquitoes at various time points using the Trizol method and subsequently detected by qRT-PCR. Positive midgut samples were selected, with three pools in each group. Ten positive midgut samples from each pool were combined, and three independent biological replicates were prepared for each pool.

Library preparation, sequencing, and data acquisition were performed by the Biomedical Public Service Platform of Jiangbei New District in Nanjing. Libraries were prepared using the VAHTS Universal V8 RNA-seq Library Prep Kit (Vazyme, CHN) and sequenced on the lllumina NovaSeq 6000 sequencing platform. Raw sequencing data were filtered by using fastp v0.23.2. The data filtering process uses parameters recommended by fastq software, followed by ribosomal RNA filtration using the sortmerna v4.3.4. The resulting filtered sequence data were aligned to the reference genome (AaegL5.0 (GCF_002204515.2)) with the STAR v2.7.10a, and quality assessment is performed using RSeQC v4.0.0, QualiMap v2.2.2, featureCounts v2.01, and Preseq v3.1.1.

The analysis results of Venn plots were generated using the CNSknowall platform (https://cnsknowall.com), a comprehensive web service for data analysis and visualization. The GO analysis was performed using g:Profiler (version e111_eg58_p18_f463989d) with g:SCS multiple testing correction method applying significance threshold of 0.05. The GO enrichment bubble diagram is made with ggplot2 of R.

*Ae. aegypti* immune-related genes downloaded from the *Ae. aegypti* transcriptome mapping article published by Hixson et al. in 2022 (49). Candidate immune-related genes satisfying |log2FoldChange| ≥ 1 and padj ≤0.05 were considered to be differentially expressed and used for subsequent plotting. The graphs were plotted using Matplotlib v1.4.1 (50) and Seaborn v0.13 (51).

### siRNA-mediated knockdown of gene expression

siRNA was obtained and purified from Sangon Biotech Company. C6/36 cell lines were seeded in 24-well plates at a density of 3 × 10^5^ cells per well. At 50% confluency, 100 nM of the targeted siRNA or control siRNA (sequence see Table S3) was transfected into cells using GenMute siRNA Transfection Reagent (SignaGen Laboratories, USA) following manufacturer’s instructions. At 24 h post- transfection, the virus was added to cells at MOI = 0.01 for 1 h. The inoculum was then discarded, and the cells were washed three times with 1 × PBS. The culture was then supplemented with fresh medium and placed in the incubator. The supernatant was collected at 24 h post-infection for qRT-PCR analysis of viral RNA levels. Three independent experiments were conducted in the experiment.

Knockout efficiency detection: at 24 h post-transfection, the culture medium was discarded, and the cells were washed three times with 1 × PBS. Cell samples were collected, and the mRNA expression level of the target gene was measured using qRT-PCR. The primers used for the analysis are listed in Table S1.

### Generation of transient cell lines

The overexpression plasmid is based on pAc5.1 as the skeleton, containing the Ac5 promoter, enhanced green fluorescent protein (EGFP), and His tag. The target gene was PCR amplified from the RNA of *Ae. aegypti* using specific primers. The target gene was inserted into EGFP by homologous recombination. The resulting constructs were cultured in *Escherichia coli* TOP 10 strain (Sangon Biotech, CHN) and subjected to sequencing for validation of the plasmid (Sangon Biotech, CHN). The specific primers are listed in Table. S1.

C6/36 cell lines were seeded in 24 well plates at a density of 3 × 10⁵ cells per well. At 50% confluency, 250 ng of the indicated overexpression plasmid or vector plasmid was transfected into the cells using the FuGENE HD Transfection Reagent (Promega, USA) in accordance with the manufacturer’s instructions. At 48 h post-transfection, the virus was added to the cells at MOI = 1 for 1 h. Subsequently, the inoculum was discarded, and the cells were washed three times with 1 × PBS. Fresh medium was then added, and the cells were incubated. The supernatant was collected at 24 h post-infection for qRT-PCR analysis of viral RNA levels. Three independent experiments were conducted in the experiment.

At 24 h post-transfection, green fluorescence expression was observed under a fluorescence microscope. Following this, the culture medium was discarded, and the cells were washed three times with 1 × PBS. Cell samples were collected and subjected to western blot analysis to assess the level of gene expression.

### Western blot

C6/36 cells were lysed in RIPA lysis buffer (Cell Signaling Technology, USA). Proteins were separated under denaturing conditions on 4%–20% polyacrylamide gels (Genscript, CHN) and transferred onto a nylon membrane (Invitrogen, USA). The membranes were blocked with 5% skim milk (Beyotime, CHN) diluted in PBST (Coolaber, CHN) at room temperature for 1 h. They were then incubated with 1:5,000 primary antibodies (6*His, His-Tag Monoclonal antibody, Proteintech, CHN) or 1:5,000 Beta Actin (MCE, USA) at 4°C overnight. Following three washes with PBST buffer, the membranes were incubated with 1:5,000 secondary antibodies (HRP-conjugated Goat Anti-Mouse IgG, Proteintech, CHN) at room temperature for 1 h. After three PBST washes, the blots were visualized using a fully automatic chemiluminescence imaging system (Tanon, CHN).

### Statistical analyses

Statistical analysis was performed using GraphPad Prism version 10.1.2. Significance was determined using the Mann-Whitney *U* test, two-way ANOVA with Šídák’s multiple comparisons test, Welch’s *t* test, and chi-square test, as specified in the figure legends. *P* values were derived from three biological replicates, unless otherwise noted.

## Data Availability

The transcriptome data are available at the Genome Sequence Archive (GSA) under Accession ID: CRA025411 (https://ngdc.cncb.ac.cn/gsa/browse/CRA025411) and BioProject ID: PRJCA039636 (https://ngdc.cncb.ac.cn/bioproject/browse/PRJCA039636). The data can be accessed publicly at https://ngdc.cncb.ac.cn/gsa/. Other data supporting the conclusions of this study are presented in the article and supplemental material.

## SUPPLEMENTAL MATERIAL

Supplemental material jvi.00758-25-s0001.docx Figures S1 to S6; Tables S1 to S3.

ASM does not own the copyrights to Supplemental Material that may be linked to, or accessed through, an article. The authors have granted ASM a non-exclusive, world-wide license to publish the Supplemental Material files. Please contact the corresponding author directly for reuse.
