# Supplementary material for: DENV-2 3′UTR dumbbell structure is a critical factor for viral infection and dissemination in Aedes mosquito
Source: J Virol. 2025 Jul 22;99(8):e00758-25. doi: 10.1128/jvi.00758-25 (PMC12363184; doi:10.1128/jvi.00758-25)
Supplement: Supplemental material — Figures S1 to S6; Tables S1 to S3. [file jvi.00758-25-s0001.docx]

**Supplementary Materials**

**
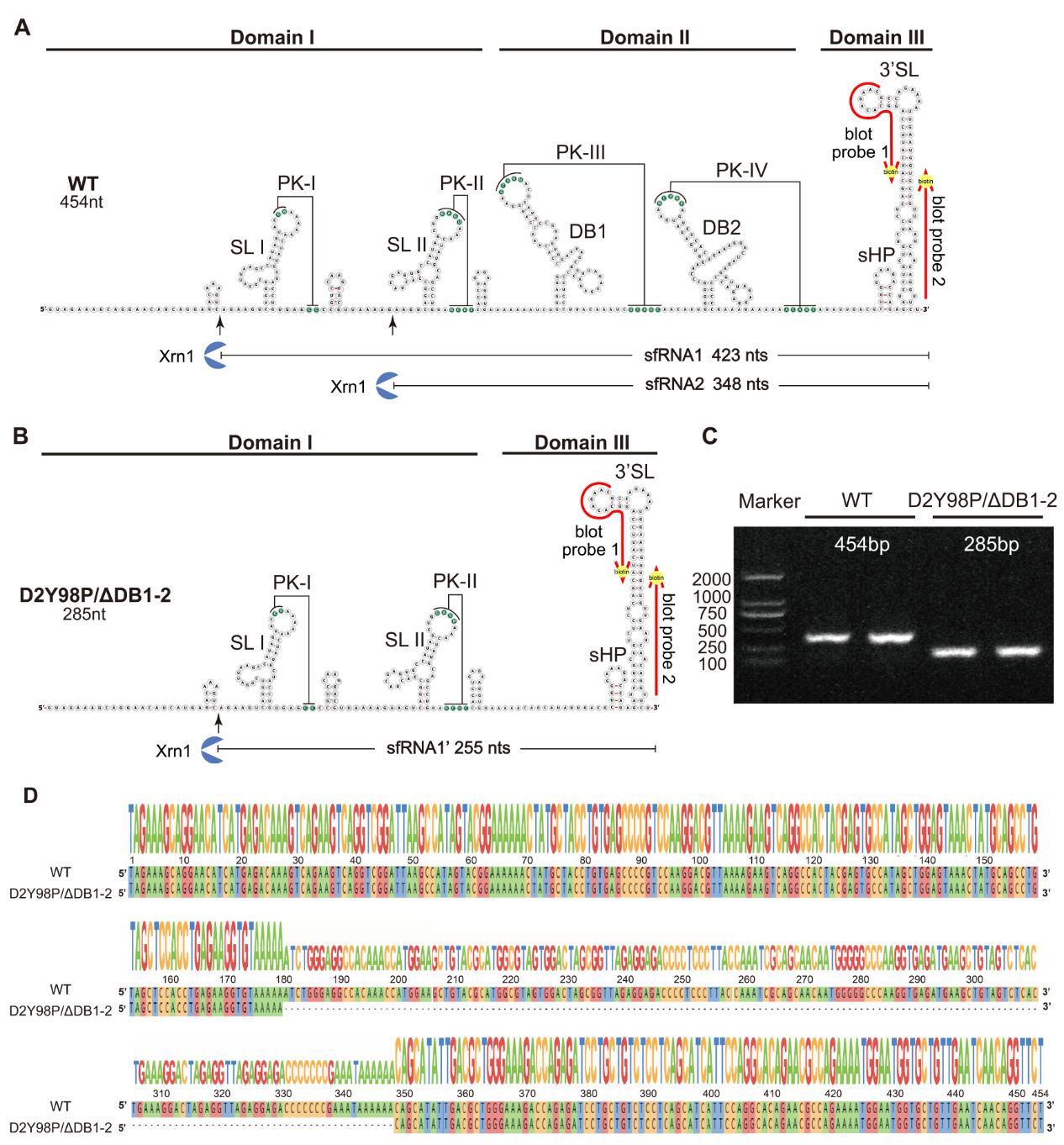
**

**Fig S1****.** **Construction of DB-deficient DENV-2.**

(A) The 3’UTR RNA secondary structural organization of wild type (WT) DENV-2. (B) The 3’UTR RNA secondary structural organization of D2Y98P/ΔDB1-2 mutant with DB structure deletion. (C) RT-PCR products for 3’UTR of WT and D2Y98P/ΔDB1-2. (D) The 3’UTR sequencing confirmation for WT and D2Y98P/ΔDB1-2. SL, stem loop structures; DB, dumbbell structures; sHP, short hairpin; 3’SL, 3’stem loop; PK, pseudoknot. The black arrow indicates the cleavage site of the Xrn1; The red arrows represent the binding sites of the northern blot probe.


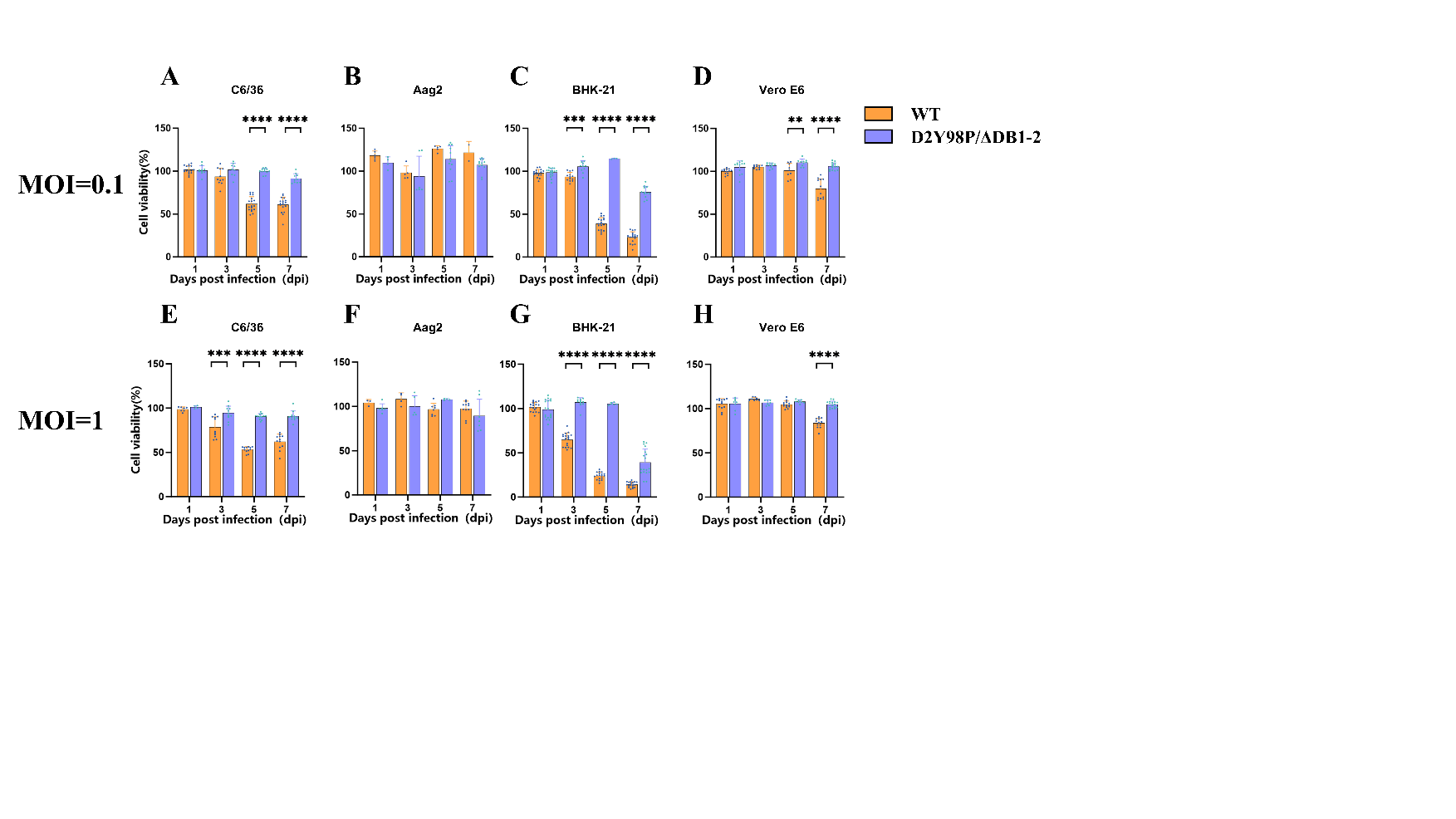


Fig S2. Assay of different cell viability.

(A-H) Cytotoxicity of WT and D2Y98P/ΔDB1-2 determined using Cell Counting Kit-8 in mosquito cell lines (C6/36 (A, E) and Aag2 (B, F)) and mammalian cell lines (BHK-21 (C, G), and Vero E6 (D, H)) at different MOIs (1 and 0.1). Data are expressed as the mean ± SD. The experiment was performed in duplicate. Statistical analysis was performed using the Mann-Whitney U test. (*: *P* ≤ 0.05, **: *P* ≤ 0.01, ***: *P* ≤ 0.001, ****: *P* ≤ 0.0001)


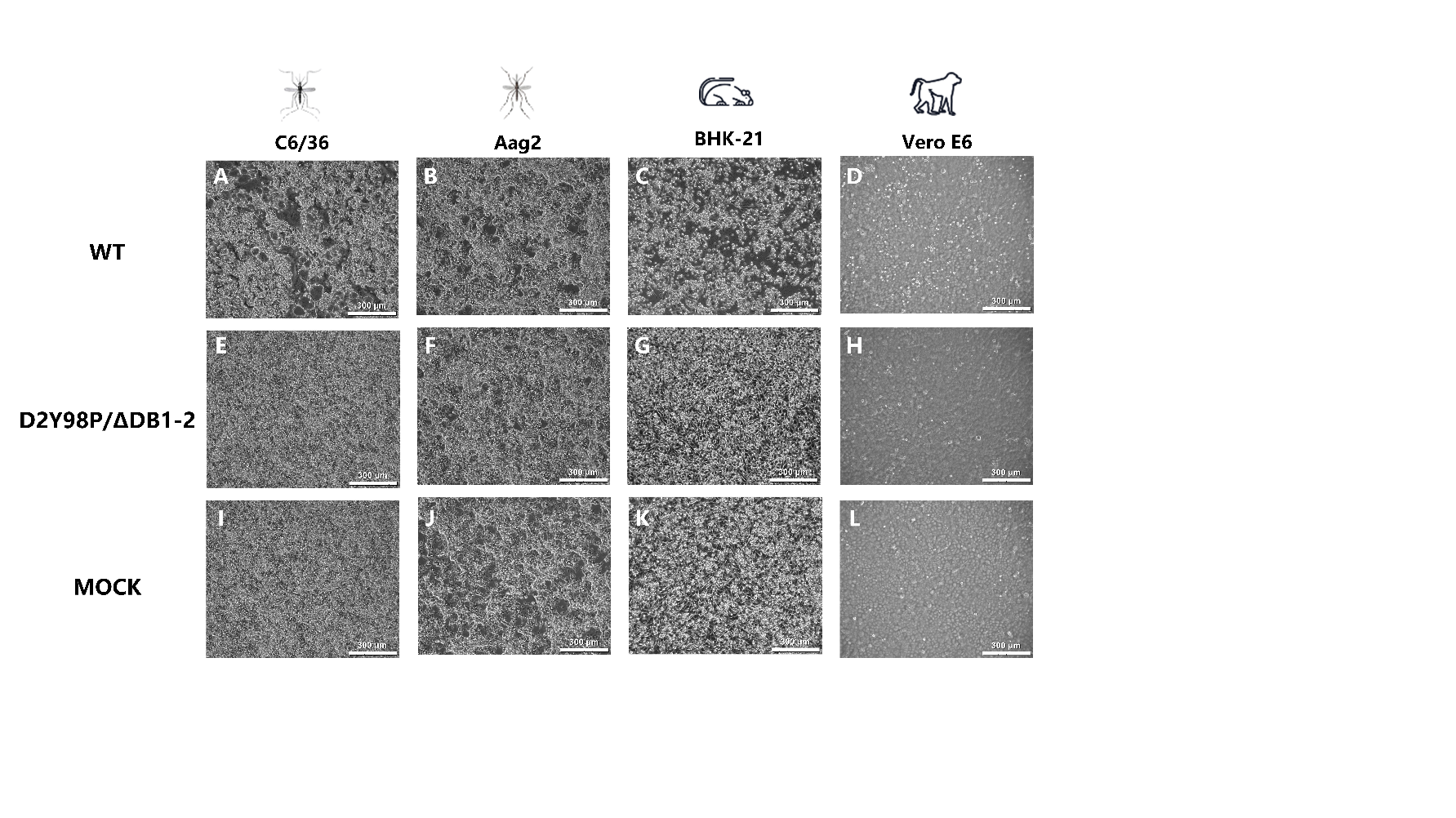


Fig S3. Cytopathic changes observed in different cell lines.

(A-D) The infection of WT in mosquito cell lines (C6/36 and Aag2) and vertebrate cell lines (BHK-21 and Vero E6). (E-H) The infection of D2Y98P/ΔDB1-2 in mosquito cell lines (C6/36 and Aag2) and vertebrate cell lines (BHK-21 and Vero E6). (I-L) Uninfected mosquito cell lines (C6/36 and Aag2) and vertebrate cell lines (BHK-21 and Vero E6). Each virus was inoculated cell lines at an MOI of 1, and observations were made at 7 d.p.i.. Morphological changes were observed using brightfield microcopy, with a scale bar of 300 μm.


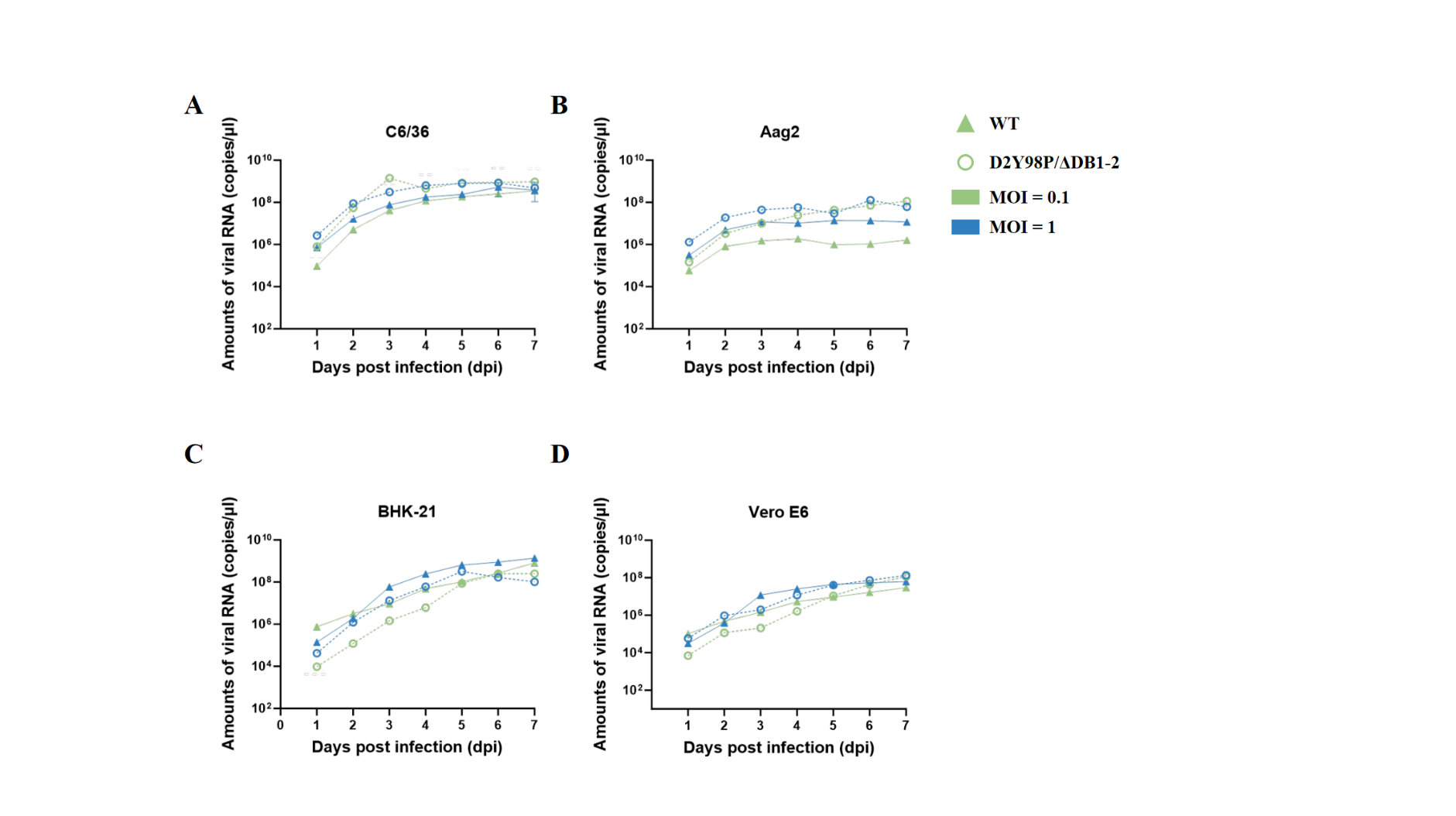


**Fig S4. Growth kinetics curves of WT and D2Y98P/ΔDB1-2 in mosquito and mammalian cell lines.**

(A-D) The viruses were inoculated into mosquito cell lines C6/36 (A) and Aag2 (B) and mammalian cell lines BHK-21 (C), and Vero E6 (D) with different MOIs. The cell supernatant was collected at every 24 h until 7 d.p.i. and detected by qRT-PCR. The solid round line represents D2Y98P and the dashed triangular line represents D2Y98P/ΔDB1-2. Blue means MOI = 1, green means MOI = 0.1.


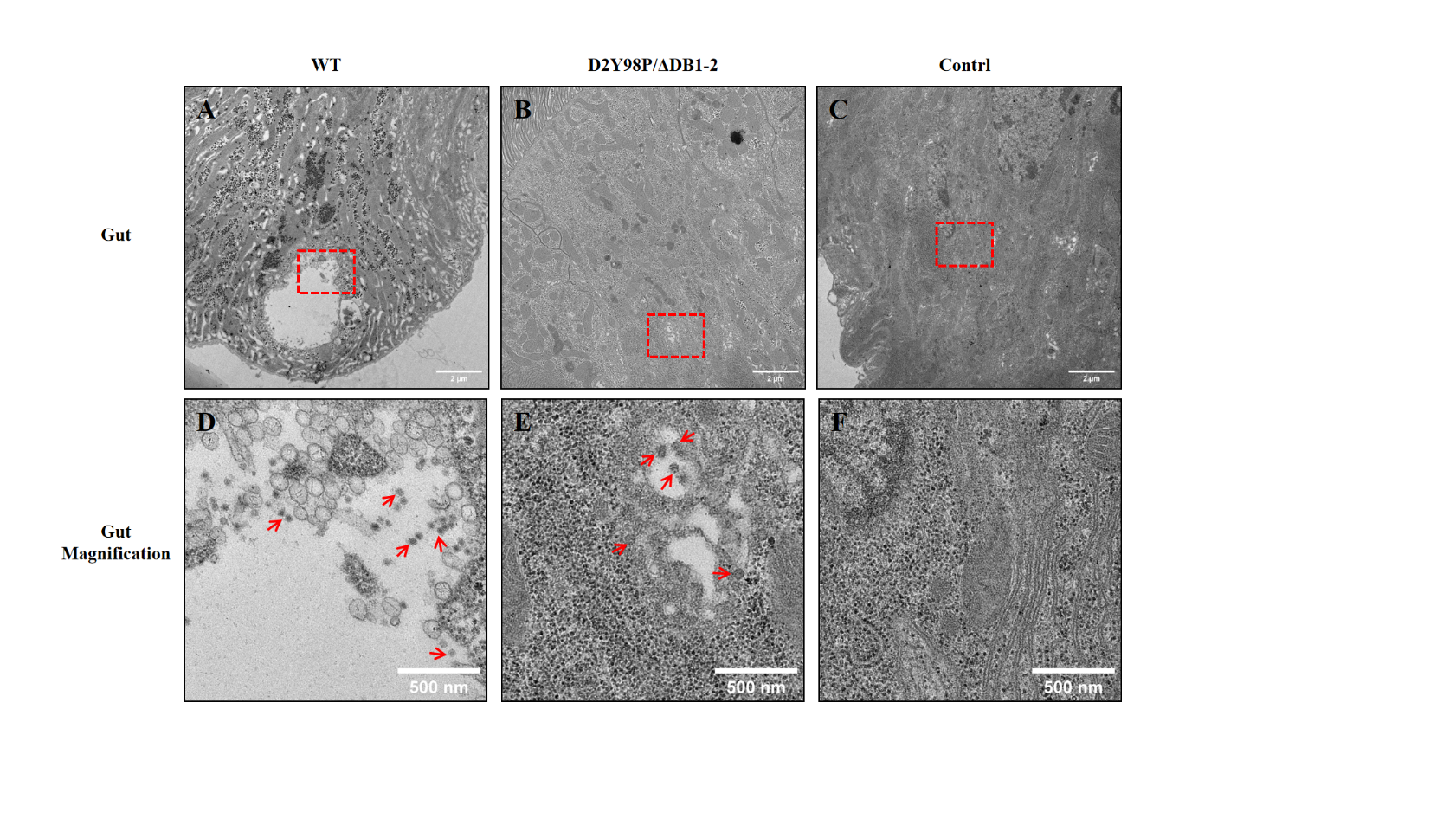


**Fig S5. Electron micrograph of viral particles in infected *Ae. aegypti* gut.**

(A-F) Ultrathin sections were made from the gut of mosquitoes at 7 d.p.i. through oral infection, and electron microscopy was used to observe the virus particles. (A) shows the gut of mosquitoes infected with WT, while (B) shows D2Y98P/ΔDB1-2. (C) represents an uninfected mosquito gut. Enlarged images of (A), (B), and (C) are shown in (D), (E), and (F) respectively, with red arrows indicating the virions.


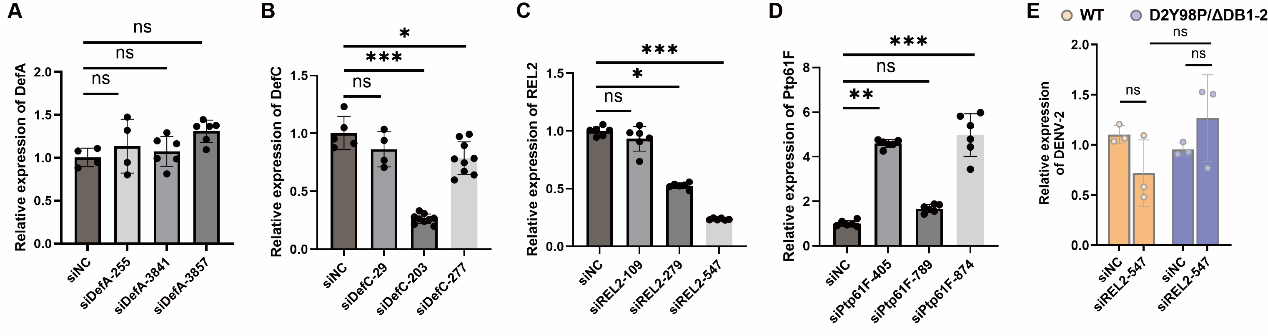


**Fig S6. siRNA-mediated knockdown on different genes.**

(A-D) Knockdown efficiency of (A) DefA, (B) DefC, (C) REL2 and (D) Ptp61F genes in C6/36 cell line was measured by qRT-PCR. Data are expressed as the mean with 95% CI from three biological replicates (n = 6 technical replicates). (E) DENV-2 replication in C6/36 cell lines after DefC knockdown. Viral replication was detected by qRT-PCR. WT is represented by orange, and D2Y98P/ΔDB1-2 by purple. Data are presented as the mean ± SD from three biological replicates (n = 5 technical replicates). All significance levels are denoted as: *: *P* ≤ 0.05, **: *P* ≤ 0.01, ***: *P* ≤ 0.001, ****: *P* ≤ 0.0001. Analyses were performed using Mann-Whitney U tests (A-D) or Welch’s t test (E).

**Table S1. All primer pairs and standard curve**

| Primer name | 5’-3’ Sequence |
| --- | --- |
| For RT-qPCR | |
| DENV2-F | TGCCCAACACAAGGRGAACC |
| DENV2-R | GCRCAGGTCACAATGCCYCC |
| DENV2-Probe | TGGTRGACAGAGGATGGGGRAATGGAT |
| G-F | CCATGAAAAGATTCAGAAGAG |
| SF-F | TGTGAGCCCCGTCCAAGGA |
| GSF-R | GGAGACAGCAGGATCTCTG |
| GSF-Probe | AAACTATGCAGCCTGTAGCTCCACC |
| For RT-PCR |  |
| 3’UTR-F | TAGAAAGCAGGAACATCATGAGACA |
| 3’UTR-R | AGAACCTGTTGATTCAACAGCACC |

**DENV-2 E gene standard curve**

Y=-3.630x+40.258


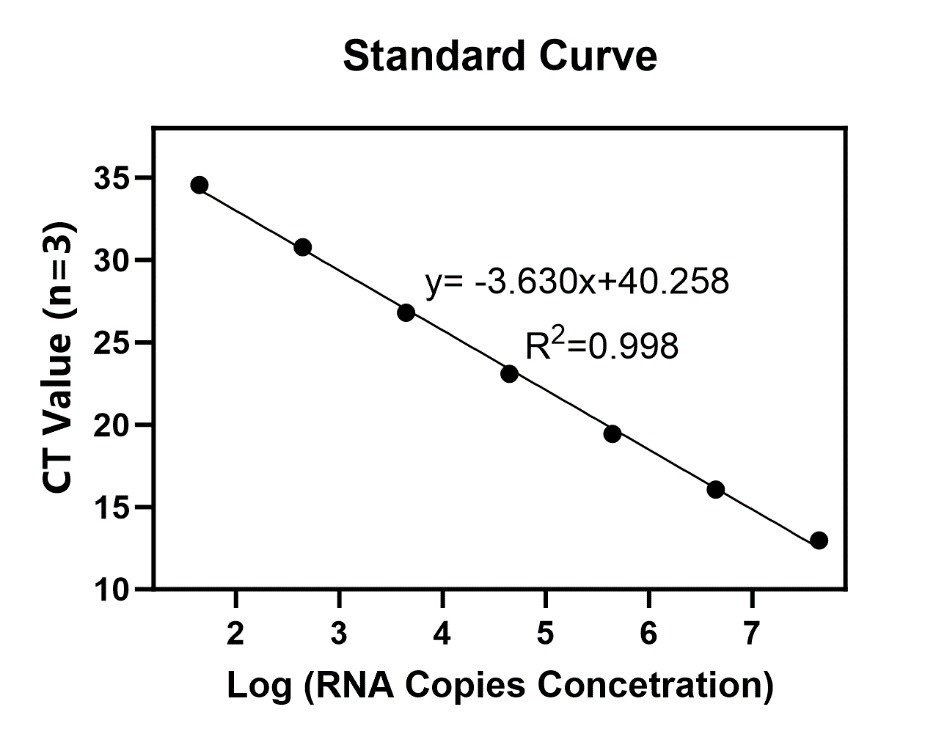
R^2^=0.998

**DENV-2 gRNA standard curve**

Y=-3.424x+49.188

R^2^=0.998

**DENV-2 sfRNA standard curve**

Y=-3.296x+47.173


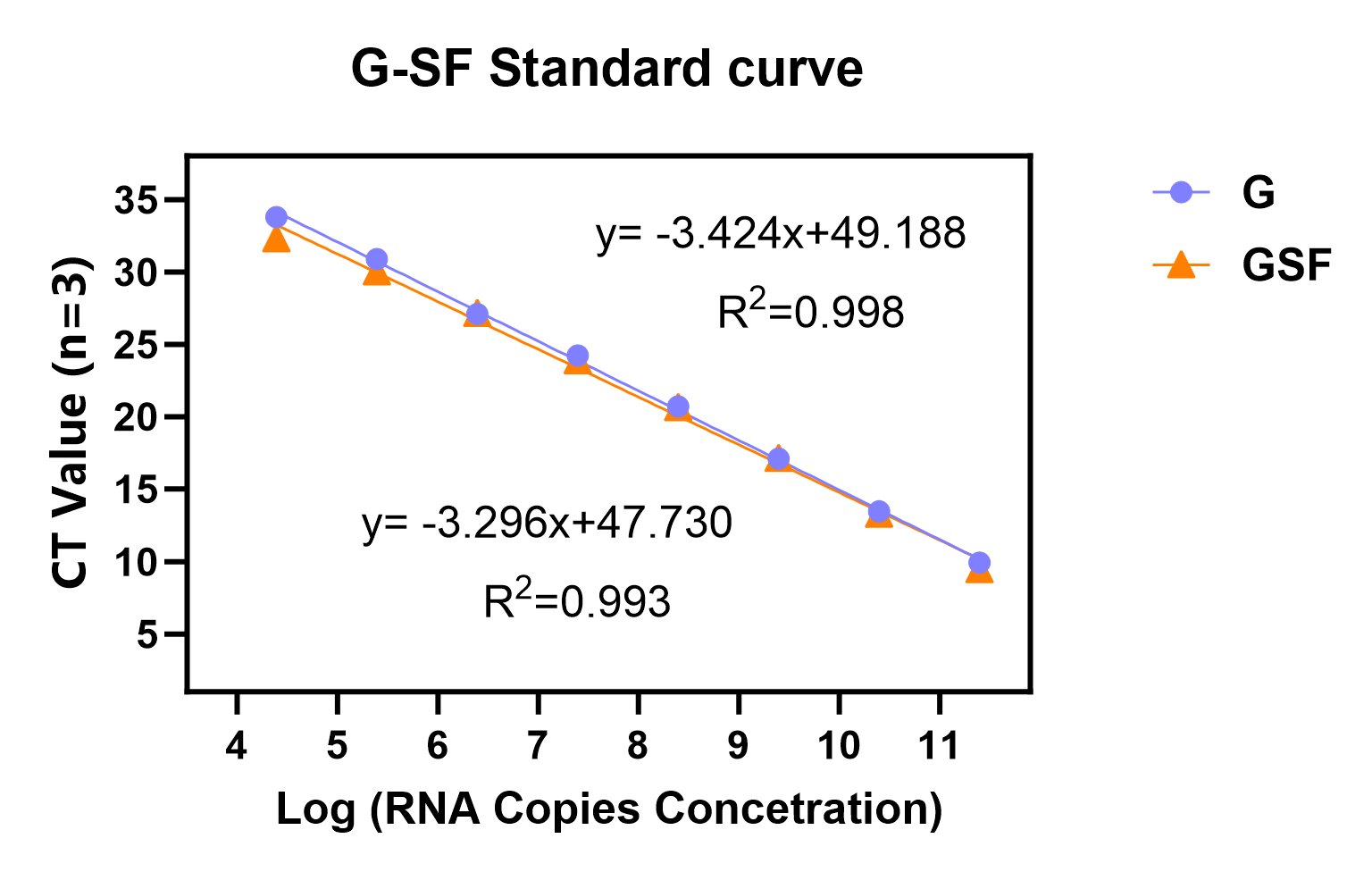
R^2^=0.993

**Table S2. The top 20 genes exhibiting differential expression.**

**The midgut differential gene set in D2Y98P/ΔDB1-2 infection group at 4 d.p.i.**

| **Gene_id** | **Log2FoldChange** | **Gene_biotype** | **Description** |
| --- | --- | --- | --- |
| LOC5576254 | 8.67 | protein_coding | 39S ribosomal protein L53%2C mitochondrial |
| LOC5564236 | 3.04 | protein_coding | GATA zinc finger domain-containing protein 10 |
| LOC5566344 | 2.36 | protein_coding | carbonic anhydrase 1%2C |
| LOC5571830 | 2.33 | protein_coding | serine protease SP24D |
| LOC5578477 | 2.32 | protein_coding | methionine synthase reductase |
| LOC5576192 | 1.89 | protein_coding | multidrug resistance-associated protein 4%2C |
| LOC5574462 | 1.89 | protein_coding | protein suppressor 2 of zeste%2C |
| LOC5578914 | 1.84 | protein_coding | glucosylceramidase |
| LOC110680956 | 1.81 | protein_coding | nose resistant to fluoxetine protein 6-like |
| LOC110679381 | 1.56 | protein_coding | alpha-1%2C2-mannosyltransferase ALG9-like |
| LOC5573499 | -2.01 | protein_coding | calcium/calmodulin-dependent 3’%2C5’-cyclic nucleotide phosphodiesterase 1 |
| LOC5570072 | -2.04 | protein_coding | coiled-coil domain-containing protein 39 |
| LOC5574622 | -2.14 | protein_coding | ATP-binding cassette sub-family G member 8%2C |
| LOC5573255 | -2.24 | protein_coding | alpha-tocopherol transfer protein |
| LOC110680917 | -2.36 | protein_coding | uncharacterized LOC110680917%2C |
| LOC5571683 | -2.39 | protein_coding | uncharacterized LOC5571683%2C |
| LOC110676173 | -2.42 | protein_coding | uncharacterized LOC110676173 |
| LOC5577908 | -2.46 | protein_coding | alpha-tocopherol transfer protein-like%2C |
| LOC5576526 | -2.46 | protein_coding | putative transporter SVOPL |
| LOC110681163 | -2.6 | protein_coding | neuropeptide FF receptor 2-like%2C |

**The midgut differential gene set in D2Y98P/ΔDB1-2 infection group at 7 d.p.i.**

| **Gene_id** | **Log2FoldChange** | **Gene_biotype** | **Description** |
| --- | --- | --- | --- |
| LOC110675944 | 6.06 | protein_coding | peritrophin-1-like |
| LOC5573022 | 3.3 | protein_coding | trypsin 5G1-like |
| LOC5577569 | 2.77 | protein_coding | collagenase |
| LOC5564288 | 2.46 | protein_coding | collectin-11 |
| LOC5570576 | 2.42 | protein_coding | uncharacterized LOC5570576 |
| LOC5572428 | 2.37 | protein_coding | CD109 antigen |
| LOC5576463 | 2.28 | protein_coding | uncharacterized LOC5576463 |
| LOC5571819 | 2.24 | protein_coding | proton-coupled folate transporter |
| LOC5579094 | 2.23 | protein_coding | defensin-C |
| LOC5568635 | 2.18 | protein_coding | putative phosphatidate phosphatase%2C |
| LOC5575231 | -4.79 | protein_coding | mRNA cap guanine-N7 methyltransferase%2C |
| LOC110681056 | -5.76 | protein_coding | WD repeat-containing and planar cell polarity effector protein fritz-like |
| LOC110680346 | -5.77 | protein_coding | HEAT repeat-containing protein 6-like |
| LOC5577277 | -6.35 | protein_coding | protein G12%2C |
| LOC5566889 | -6.45 | protein_coding | protein-cysteine N-palmitoyltransferase Rasp%2C |
| LOC110681042 | -7.38 | protein_coding | mitochondrial import receptor subunit TOM40 homolog 1-like |
| LOC110680221 | -7.66 | protein_coding | nicalin-1-like |
| LOC5574479 | -19.04 | protein_coding | gamma-secretase subunit Aph-1 |
| LOC5572736 | -21.88 | protein_coding | U4/U6 small nuclear ribonucleoprotein Prp4 |
| LOC5572121 | -35.61 | protein_coding | cytochrome c oxidase assembly factor 3%2C mitochondrial |

**The midgut differential gene set in D2Y98P/ΔDB1-2 infection group at 10 d.p.i.**

| **Gene_id** | **Log2FoldChange** | **Gene_biotype** | **Description** |
| --- | --- | --- | --- |
| LOC110676852 | 19.2 | protein_coding | glutathione S-transferase 1-like |
| LOC110680759 | 9.54 | protein_coding | defensin-A |
| LOC5579094 | 4.56 | protein_coding | defensin-C |
| LOC5578028 | 4.27 | protein_coding | attacin-B |
| LOC5576463 | 3.73 | protein_coding | uncharacterized LOC5576463 |
| LOC23687443 | 3.62 | protein_coding | CD109 antigen%2C |
| LOC5572428 | 3.22 | protein_coding | CD109 antigen |
| LOC5569396 | 3.18 | protein_coding | trypsin 5G1-like |
| LOC5575054 | 2.91 | protein_coding | plasma kallikrein |
| LOC5575329 | 2.78 | protein_coding | tryptase-2%2C |
| LOC5565109 | -3.11 | protein_coding | uncharacterized LOC5565109%2C |
| LOC5566288 | -3.23 | protein_coding | RNA-binding protein Musashi homolog Rbp6%2C |
| LOC5573748 | -3.9 | protein_coding | sodium- and chloride-dependent glycine transporter 2 |
| LOC5565692 | -5.15 | protein_coding | endocuticle structural glycoprotein SgAbd-2 |
| LOC5570460 | -5.82 | protein_coding | uncharacterized LOC5570460 |
| LOC110676644 | -5.9 | protein_coding | uncharacterized LOC110676644%2C |
| LOC5574481 | -6.53 | protein_coding | ankyrin repeat%2C PH and SEC7 domain containing protein secG |
| LOC5570300 | -7.05 | protein_coding | uncharacterized LOC5570300%2C |
| LOC110681160 | -8.88 | protein_coding | RNA polymerase-associated protein CTR9 homolog |
| LOC110681342 | -21.29 | protein_coding | histone H1B-like |

**The midgut differential gene set in D2Y98P/ΔDB1-2 infection group at 14 d.p.i.**

| **Gene_id** | **Log2FoldChange** | **Gene_biotype** | **Description** |
| --- | --- | --- | --- |
| LOC5565191 | 22.25 | protein_coding | pyruvate dehydrogenase [acetyl-transferring]-phosphatase 1%2C mitochondrial |
| LOC110681279 | 8.37 | protein_coding | ankyrin repeat%2C PH and SEC7 domain containing protein secG-like |
| LOC5573927 | 5.61 | protein_coding | fatty acid synthase |
| LOC5568416 | 4.67 | protein_coding | cytochrome P450 4g15 |
| LOC5564064 | 3.26 | protein_coding | cytochrome P450 4g15 |
| LOC110674776 | 2.98 | protein_coding | struthiocalcin-2-like |
| LOC5569107 | 2.65 | protein_coding | uncharacterized LOC5569107 |
| LOC5570480 | 2.64 | protein_coding | uncharacterized LOC5570480 |
| LOC5571830 | 2.59 | protein_coding | serine protease SP24D |
| LOC5570483 | 2.36 | protein_coding | acidic leucine-rich nuclear phosphoprotein 32 family member A |
| LOC5576245 | -2.45 | protein_coding | tubulin beta-3 chain |
| LOC5579424 | -2.95 | protein_coding | uncharacterized LOC5579424 |
| LOC110677665 | -3.29 | protein_coding | uncharacterized LOC110677665%2C |
| LOC5570856 | -3.4 | protein_coding | stress-activated protein kinase JNK |
| LOC5563591 | -3.5 | protein_coding | phosphoenolpyruvate carboxykinase [GTP] |
| LOC5574481 | -7.38 | protein_coding | ankyrin repeat%2C PH and SEC7 domain containing protein secG |
| LOC110676362 | -11.42 | protein_coding | UDP-glucuronosyltransferase 2B18-like |
| LOC110680759 | -21.45 | protein_coding | defensin-A |
| LOC110681535 | -21.86 | protein_coding | DNA-directed RNA polymerase III subunit RPC3-like |
| LOC110680664 | -34.73 | protein_coding | uncharacterized LOC110680664 |

**Table S3. siRNA sequence**

| **siRNA** | **Sense Strand (5’-3’)** | **Antisense Strand (5’-3’)** |
| --- | --- | --- |
| siDefA-255 | GCUACUGCAACUCCAAGAATT | UUCUUGGAGUUGCAGUAGCTT |
| siDefA-3841 | CGAUUAUCACAUCAUUCAATT | UUGAAUGAUGUGAUAAUCGTT |
| siDefA-3857 | GCUACUGCAACUCCAAGAATT | UUCUUGGAGUUGCAGUAGCTT |
| siDefC-29 | GCUCUUUGCCUCAGUGCAATT | UUGCACUGAGGCAAAGAGCTT |
| siDefC-203 | GGUGUAGGAGAUAGUGCUUTT | AAGCACUAUCUCCUACACCTT |
| siDefC-277 | GGUUUGCGUUUGUCGAAAUTT | AUUUCGACAAACGCAAACCTT |
| siREL2-109 | CCGACACUAGCAAAUACUATT | UAGUAUUUGCUAGUGUCGGTT |
| siREL2-279 | GGUCAACUGCAGACCUUUATT | UAAAGGUCUGCAGUUGACCTT |
| siREL2-547 | CGCAUAAUCUGGUGAUCAATT | UUGAUCACCAGAUUAUGCGTT |
| siPtp61F-405 | GGGCGAUACUGAUGCUGAATT | UUCAGCAUCAGUAUCGCCCTT |
| siPtp61F-789 | GUCUAGUGUUGAUCGAUAATT | UUAUCGAUCAACACUAGACTT |
| siPtp61F-874 | GAUACAAACAGUAGAUCAATT | UUGAUCUACUGUUUGUAUCTT |
